# Supplementary material for: A Linear Diubiquitin-Based Probe for Efficient and Selective Detection of the Deubiquitinating Enzyme OTULIN
Source: Cell Chem Biol. 2017 Oct 19;24(10):1299–1313.e7. doi: 10.1016/j.chembiol.2017.08.006 (PMC5658516; doi:10.1016/j.chembiol.2017.08.006)
Supplement: Document S1. Figures S1–S5 and Tables S1 and S2 [file mmc1.pdf]

Cell Chemical Biology, Volume *24*

Supplemental Information

A Linear Diubiquitin-Based Probe for Efficient and Selective Detection of the Deubiquitinating Enzyme OTULIN

Aurelia Weber, Paul R. Elliott, Adan Pinto-Fernandez, Sarah Bonham, Benedikt M. Kessler, David Komander, Farid El Oualid, and Daniel Krappmann

SUPPLEMENTARY TABLES AND FIGURES

A linear diubiquitin-based probe for efficient and selective detection of the deubiquitinating enzyme OTULIN

Aurelia Weber, Paul R. Elliott, Adan Pinto-Fernandez, Sarah Bonham, Benedikt M. Kessler, David Komander, Farid El Oualid, and Daniel Krappmann

Values in parentheses are for the highest resolution shell.

	OTULIN 80-352-biotin-Ahx-Ub(1-75)-Dha-Ub
Data collection	
Beamline	Diamond I04
Space group	*H* 3_2
a, *b*, *c* (Å)	101.14, 101.14, 277.92
α, β, γ (°)	90.00, 90.00 ,120.00
Wavelength	0.9795
Resolution (Å)	54.43-2.95 (3.13-2.95)
R_{merge}	5.9 (70.3)
$< I / \sigma I >$	13.6 (2.0)
CC(1/2)	0.99 (0.91)
Completeness (%)	100 (100)
Redundancy	6.4 (6.5)
Refinement	
Resolution (Å)	92.64-2.95
No. reflections	11911
R_{work} / R_{free}	0.22/0.32
No. atoms	3104
Protein	3104
Ligand/ion	-
Water	-
B-factors	
Wilson B	93.98
Protein	77.08
Ligand/ion	-
Water	-
R.m.s deviations	
Bond lengths (Å)	0.018
Bond angles (°)	1.955
Ramachandran statistics (outliers, allowed, favoured)	

Dataset was collected from a single crystal.

Table S1 (related to Fig. 3): Data collection and refinement statistics for crystallography.

		LOG2 LFQ intensities																					
		No Probe				OTULIN-APB				OTULIN-ABP + Apyrase				OTULIN-ABP + Apyrase + PR619				Significance difference No Probe_OTULIN-ABP (Student's T-test)					
**Protein IDs**	**Protein names**	**LFQ I 1_1**	**LFQ I 1_2**	**LFQ 1_3**	**LFQ I 1_4**	**LFQ I 2_1**	**LFQ I 2_2**	**LFQ I 2_3**	**LFQ I 2_4**	**LFQ 3_1**	**LFQ I 3_2**	**LFQ I 3_3**	**LFQ I 3_4**	**LFQ I 4_1**	**LFQ I 4_2**	**LFQ I 4_3**	**LFQ I 4_4**		**N: -Log STT**	**N: STT**	**N: STT statistic**	**MW [kDa]**	**Score**
P22314-2;P22314	UBA1	17,7766	17,5063	17,5048	17,707	29,2205	29,5495	29,7971	30,1985	23,5485	21,5931	19,6165	20,4098	21,8335	20,1046	20,6147	19,3192	+	8,63627	-12,0677	-5,4417	113,8	323,31
Q96BN8;H0Y9T0	OTULIN	17,1191	16,7217	15,4617	16,6438	29,0197	29,4706	29,7409	29,2958	29,8383	29,3178	28,8216	28,1979	23,6984	23,434	22,1332	22,3256	+	7,30689	-12,8952	-5,40014	40,262	308,75
P15374;Q5TBK7	UCHL3	17,7447	17,6595	15,7178	15,9059	24,2089	25,0379	24,8467	24,4435	25,0074	25,0494	24,3842	24,4533	25,1512	25,7674	24,9247	24,739	+	5,01022	-7,87727	-3,05465	26,182	62,597
A0AVT1;A0AVT1-2	UBA6	18,5863	16,5304	17,4451	17,8637	22,7336	23,4294	24,0412	24,2625	17,2079	18,4227	17,7002	16,9856	18,042	17,8221	17,6305	17,2594	+	4,4604	-6,0103	-2,35751	117,97	75,662
F8VSA6;E9PS38	NEDD8	17,8619	17,5603	15,351	15,947	22,6509	23,4459	24,6994	23,244	23,8412	23,2567	23,1742	23,0965	24,4478	24,3355	24,1069	24,2666	+	4,0151	-6,82999	-2,48599	5,8668	21,287
P45974-2;P45974	USP5	17,798	20,1282	15,5395	20,1224	26,2123	27,7202	26,3578	26,3365	27,9555	26,4718	26,66	26,1792	26,3028	26,0168	25,6588	25,687	+	3,42288	-8,25972	-2,61767	93,307	154,3
F5H265;J3QS39	UBC;UBB	23,6918	24,1324	16,9545	16,6629	29,8302	30,9425	31,9719	31,6184	30,5698	31,2093	30,1123	30,8753	30,7342	30,8444	29,8138	31,1985	+	2,65046	-10,7303	-2,61282	16,841	323,31

Table S2 (related to Fig. 6): DUB enzymes identified as OTULIN ABP interactors by LC-MS/MS after biotin-PD. Perseus Label-free interaction data analysis workflow was performed using the following cutoff parameters: FDR = 0.01 and s0 = 2. LFQ intensities are given for each sample. Significance was calculated by Student's T test (STT).

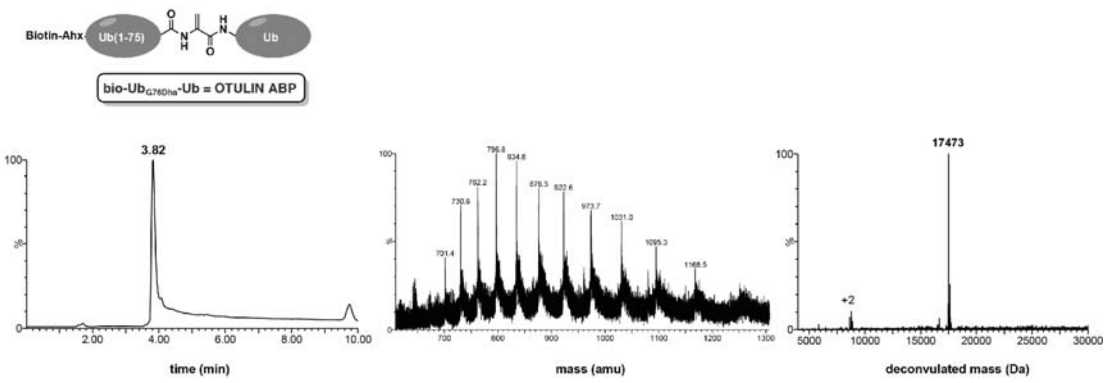

Figure S1 (related to Fig. 1): LC-MS analysis Biotin-Ahx-Ub(1-75)-Dha-Ub (bio-Ub$_{G76Dha}$-Ub).
Mobile phase A = 1% CH_3CN, 0.1% formic acid in water (milliQ) and B = 1% water (milliQ) and 0.1% formic acid in CH_3CN. XBridge BEH300 C18 5µm 4.6x100mm; column T = 40°C, flow= 0.8 mL/min. Gradient: 0 - 1.5 min: 5-30% B; 1.5 - 8 min: 30-50% B; 8 – 9.5 min: 50-95% B.

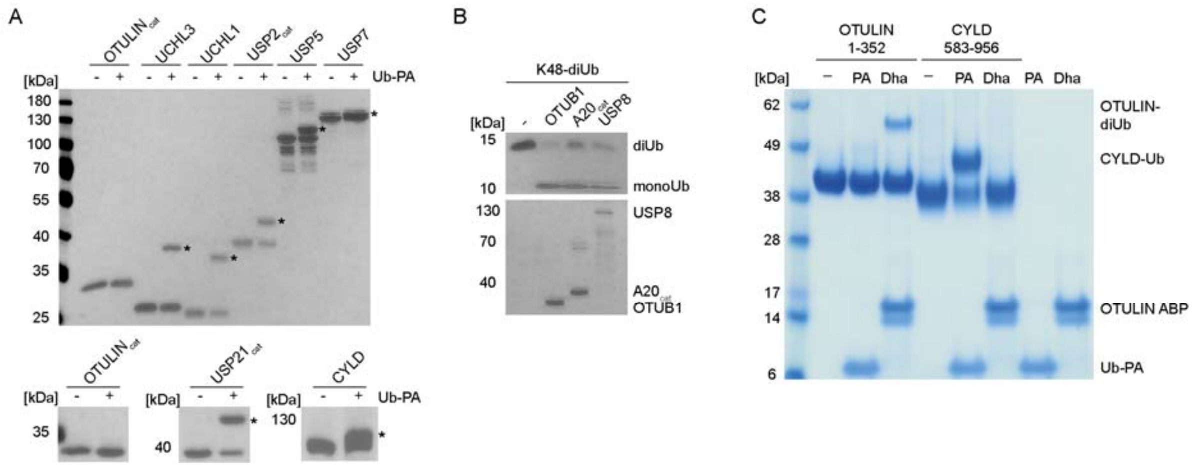

Figure S2 (related to Figure 4): Validation of DUB activities

A) Validation of DUB activities with Ub-PA probe. Recombinant DUBs (0.5 µM) were incubated at 30°C with biotin-Ahx-Ub-PA probe (5 µM) for 1 h. Labeling was monitored by Silver staining. Asterisks denote the DUB-Ub adducts.

B) Validation of DUB activities by K48-diUb cleavage assay. OTUB1, $A20_{cat}$ and USP8 (0.5 µM) were incubated with 500 ng of K48-linked diUb at 30°C for 1 h before analyzing the diUb cleavage by Silver staining.

C) Direct comparison of OTULIN and CYLD labeling by OTULIN ABP and Ub-PA. Recombinant OTULIN (aa 1-352) or CYLD (aa 583-956), both at 30 µM were mixed with equimolar Ub-PA (PA) or bio-Ub_{G76Dha}-Ub (Dha) and incubated at RT for 1 h. Samples were resolved by SDS-PAGE and stained with Coomassie.

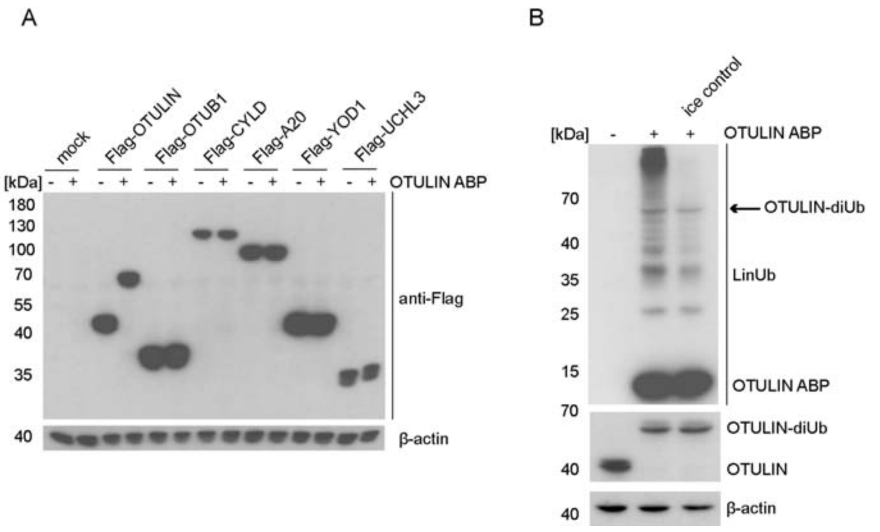

Figure S3 (related to Fig. 5): Labeling of overexpressed DUBs and induced Ub chain synthesis by OTULIN ABP.

A) Cross-reactivity of OTULIN ABP with transfected DUBs in HEK293 cells. Cell extracts from HEK293 cells overexpressing Flag-tagged DUBs (~$2.5*10^5$ cells / reaction) were incubated with OTULIN ABP (30°C, 1 h). Labeling of DUBs was analyzed by Western Blot using anti-Flag antibody.

B) Temperature-dependent polyUb chain formation induced by OTULIN ABP. HEK293 cell extracts (~$2.5*10^5$ cells / reaction) were mixed with 1 µg OTULIN ABP and incubated for 30 min at 30°C. In the control lane, the enzymatic reaction was put on ice after an initial 2 min incubation at 30°C. Formation of the OTULIN-diUb adducts and linear ubiquitin chains were monitored by Western Blot.

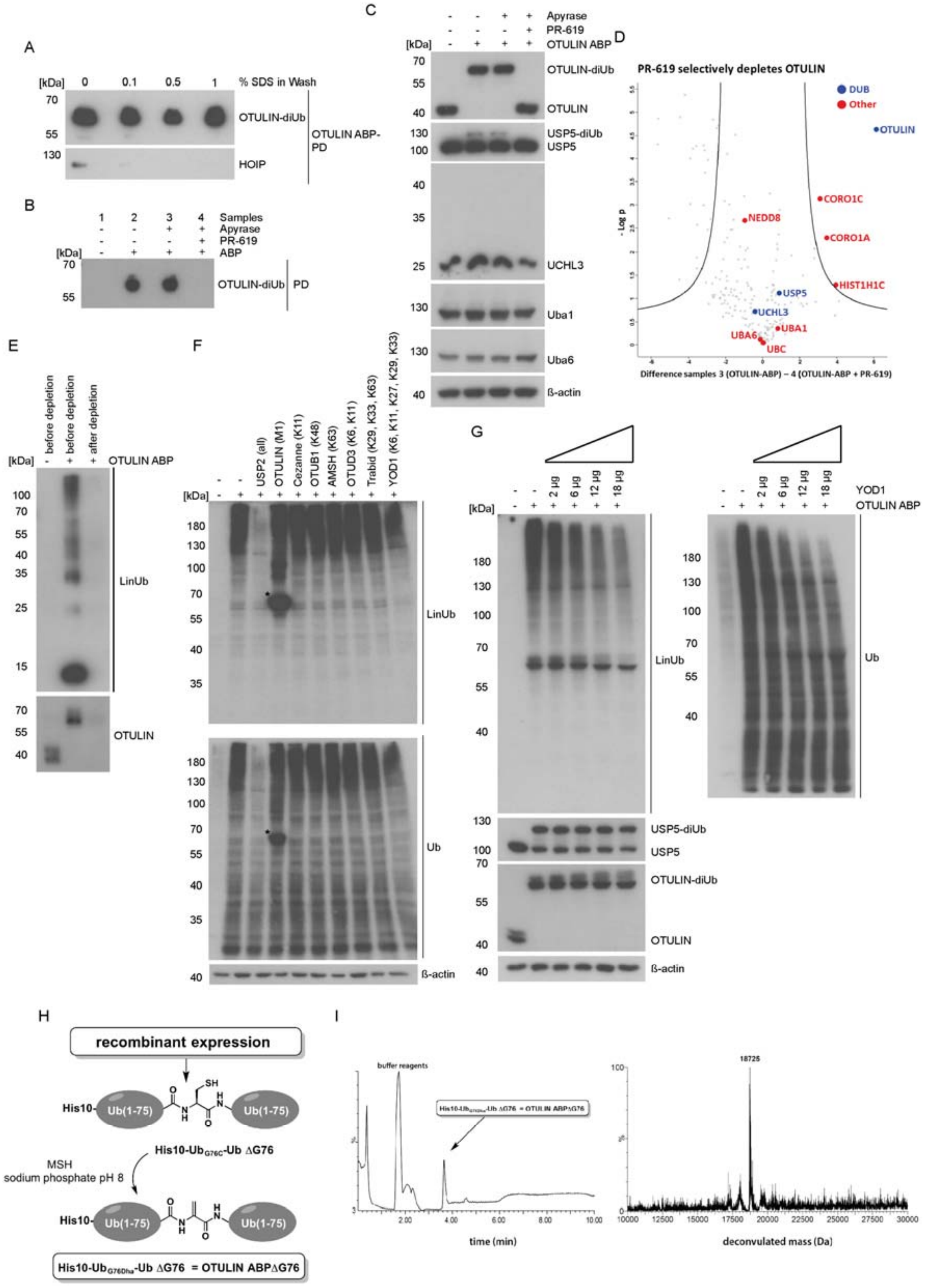

Figure S4 (related to Fig. 6): Specificity of probe labeling and auto-conjugation; synthesis of OTULIN ABPΔG76

A) Biotin-PD of OTULIN-diUb complex under high stringent washing conditions. 4 µg of OTULIN ABP were incubated in extracts of Jurkat T cells ($18*10^6$ cells per reaction) for 15 min at RT. After PD, beads were washed twice with increasing SDS concentrations to enrich for covalent ABP interactors. Pull-down eluates were analyzed for OTULIN-diUb complex and HOIP by Western Blot.

B) Control of OTULIN-diUb pull-down in samples before LC-MS/MS analyses. Cell extracts of $5x10^7$ Jurkat T cells were subjected to treatments (as indicated; see Fig. 6A) and biotin-PD including 1% SDS washes was performed. PD of OTULIN-diUb complex was verified by analyzing 5% of the eluted material that was subsequently used for LC-MS/MS analyses.

C) OTULIN ABP labeling in extracts of Jurkat T cells. Extracts of Jurkat T cells were treated equivalent to LC-MS/MS (see Fig. 1A) and coupling of OTULIN ABP to DUBs and E1 enzymes in the extracts was determined by Western Blot.

D) Interactome analyses reveal selective depletion of OTULIN and HOIP from the OTULIN ABP by PR-619 treatment. Volcano plot demonstrates loss of binding of DUBs (blue) and other proteins (red) between control (sample 3) and PR-619 (sample 4) treatments before OTULIN ABP incubation and biotin-PD. Curves depict significant enrichment or depletion, respectively. Loss of COR1C, FHL2 and TPM3 is only associated with PR-619 treatment and not with specific OTULIN ABP interaction.

E) OTULIN ABP is auto-conjugated in cell extracts. Extracts of Jurkat T cells ($6*10^5$ cells / reaction) treated with 1 µg OTULIN ABP (45 min, 30°C) were subjected to biotin-PD. Depletion of Ub chains was demonstrated by comparing presence of Ub chains in the extracts before and after biotin-PD. Depletion of OTULIN-diUb adduct is shown as control.

F) OTULIN ABP induces generation of Ub chains that are prone to USP2 digestion. Linkage analyses of Ub chains formed in extracts of HEK293 cells after OTULIN ABP incubation (30 min at 37°C) were performed by UbiCrest digestion. Treatment with linkage-specific DUBs was performed for another 30 min. Ubiquitin chains were detected by Western Blotting with anti-M1-polyUb or anti-Ub antibodies. Asterisk indicates formation OTULIN-diUb adduct by the OTULIN ABP.

G) High concentrations of YOD1 lead to partial digestion of OTULIN ABP induced Ub chains. Increasing YOD1 amounts were added (30 min) to extracts of HEK293 cells Ub chains were generate by OTULIN ABP incubation as in D. Formation of polyUb chains was detected by Western Blotting with anti-M1-polyUb or anti-Ub antibodies.

H) Chemical synthesis of His-Ub_{G76Dha}-$Ub_{\Delta G76}$ (OTULIN ABP∆G76)

I) LC-MS analysis His-Ub(1-75)-Dha-Ub(1-75) (His-Ub_{G76Dha}-$Ub_{\Delta G76}$)

Figure S5 (related to Fig. 7)

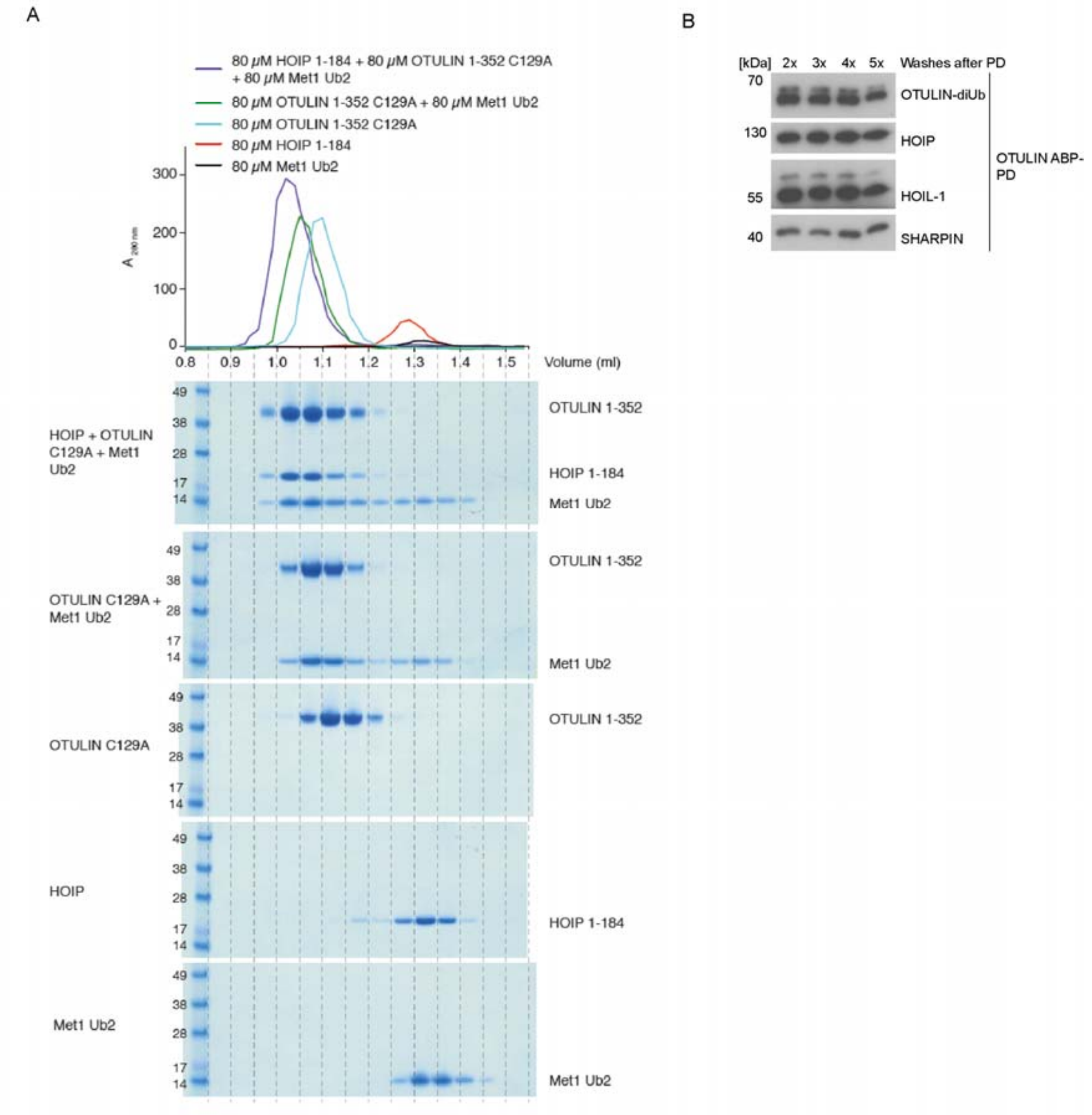

Figure S5 (related to Fig. 7): Data on stability of ternary OTULIN-diUb-LUBAC interaction *in vitro* and from cells

A) Size exclusion chromatography demonstrates ternary complex of HOIP, OTULIN and M1-linked diUb in vitro. Analytical size exclusion chromatography profiles for OTULIN (aa 1-352; C129A; cyan), HOIP PUB domain (aa 1-1184; red), M1-diUb (black curve), and the dimeric complex between OTULIN and M1-diUb (green curve) and trimeric complex between HOIP, OTULIN and M1-diUb (blue curve) at equimolar ratios. Coomassie-stained SDS-PAGE gels below show protein-containing fractions.

B) Stability of the OTULIN-diUb product bound to the cellular LUBAC in extracts. Extracts of Jurkat T cells were incubated with OTULIN ABP and biotin-PDs were performed as in Fig. 7C. Stability of the LUBAC association to the OTULIN-diUb product was monitored by analyzing successive (2x – 5x) washing steps of the pull-downs.
