# Supplementary material for: A Linear Diubiquitin-Based Probe for Efficient and Selective Detection of the Deubiquitinating Enzyme OTULIN
Source: Cell Chem Biol. 2017 Oct 19;24(10):1299–1313.e7. doi: 10.1016/j.chembiol.2017.08.006 (PMC5658516; doi:10.1016/j.chembiol.2017.08.006)
Supplement: Document S2. Article plus Supplemental Information [file mmc2.pdf]

Resource

Cell Chemical Biology

# A Linear Diubiquitin-Based Probe for Efficient and Selective Detection of the Deubiquitinating Enzyme OTULIN

## Graphical Abstract

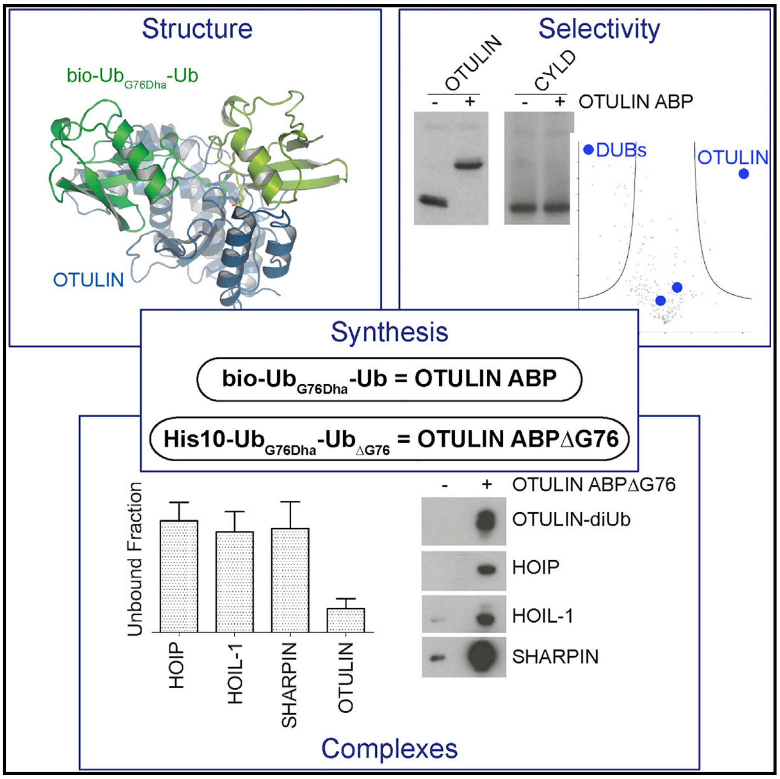


## Authors

Aurelia Weber, Paul R. Elliott, Adan Pinto-Fernandez, ..., David Komander, Farid El Oualid, Daniel Krappmann

## Correspondence

farideloualid@ubiqbio.com (F.E.O.), daniel.krappmann@helmholtz-muenchen.de (D.K.)

## In Brief

Weber et al. report on the chemical synthesis of highly selective OTULIN activity-based probes (ABPs) useful for studying enzymatic activity and cellular functions of the M1-linkage-specific ubiquitin hydrolase OTULIN.

## Highlights

- Chemical synthesis of the OTULIN ABPs Ub$_{G76Dha}$-Ub and Ub$_{G76Dha}$-Ub$_{\Delta G76}$
- OTULIN ABPs display high selectivity for labeling OTULIN
- Chemical tool to monitor OTULIN activity and interactions in cells
- Substrate-bound OTULIN associates with cellular LUBAC

Weber et al., 2017, Cell Chemical Biology *24*, 1299–1313
October 19, 2017 © 2017 MRC Laboratory of Molecular Biology. Published by Elsevier Ltd.
http://dx.doi.org/10.1016/j.chembiol.2017.08.006

CrossMark
CellPress

Cell Chemical Biology
Resource

CellPress

# A Linear Diubiquitin-Based Probe for Efficient and Selective Detection of the Deubiquitinating Enzyme OTULIN

Aurelia Weber,[1] Paul R. Elliott,[2] Adan Pinto-Fernandez,[3] Sarah Bonham,[3] Benedikt M. Kessler,[3] David Komander,[2] Farid El Oualid,[4,*] and Daniel Krappmann[1,5,*]
[1]Research Unit Cellular Signal Integration, Institute of Molecular Toxicology and Pharmacology, Helmholtz Zentrum München - German Research Center for Environmental Health, Ingolstaedter Landstrasse 1, 85764 Neuherberg, Germany
[2]Medical Research Council Laboratory of Molecular Biology, Francis Crick Avenue, Cambridge CB2 0QH, UK
[3]Target Discovery Institute, Nuffield Department of Medicine, University of Oxford, Roosevelt Drive, Oxford OX3 7FZ, UK
[4]UbiQ Bio BV, Science Park 408, 1098 XH Amsterdam, the Netherlands
[5]Lead Contact
*Correspondence: faridelouali d@ubiqbio.com (F.E.O.), daniel.krappmann@helmholtz-muenchen.de (D.K.)
http://dx.doi.org/10.1016/j.chembiol.2017.08.006

## SUMMARY

**The methionine 1 (M1)-specific deubiquitinase (DUB) OTULIN acts as a negative regulator of nuclear factor κB signaling and immune homeostasis. By replacing Gly76 in distal ubiquitin (Ub) by dehydroalanine we designed the diubiquitin (diUb) activity-based probe $Ub_{G76Dha}$-Ub (OTULIN activity-based probe [ABP]) that couples to the catalytic site of OTULIN and thereby captures OTULIN in its active conformation. The OTULIN ABP displays high selectivity for OTULIN and does not label other M1-cleaving DUBs, including CYLD. The only detectable cross-reactivities were the labeling of USP5 (Isopeptidase T) and an ATP-dependent assembly of polyOTULIN ABP chains via Ub-activating E1 enzymes. Both cross-reactivities were abolished by the removal of the C-terminal Gly in the ABP's proximal Ub, yielding the specific OTULIN probe $Ub_{G76Dha}$-$Ub_{\Delta G76}$ (OTULIN ABPΔG76). Pull-downs demonstrate that substrate-bound OTULIN associates with the linear ubiquitin chain assembly complex (LUBAC). Thus, we present a highly selective ABP for OTULIN that will facilitate studying the cellular function of this essential DUB.**

## INTRODUCTION

By assembling covalent chains with distinct linkages, the post-translational modifier ubiquitin (Ub) controls nearly all aspects of cell biology and cellular homeostasis (Komander and Rape, 2012). Conjugation of methionine 1 (M1)-linked polyubiquitin (polyUb) is catalyzed by the linear ubiquitin chain assembly complex (LUBAC), consisting of HOIP/RNF31, HOIL-1, and SHARPIN (Fiil and Gyrd-Hansen, 2014; Iwai et al., 2014). Upon stimulation, LUBAC is recruited to immune receptors, e.g., tumor necrosis factor receptor (TNFR), interleukin-1 receptor, and Toll-like receptors, where it decorates signaling mediators such as RIPKs, MYD88, IRAKs, and NEMO with M1-linked Ub chains to facilitate nuclear factor κB (NF-κB)-dependent immune and inflammatory responses. In turn, LUBAC activity needs to be tightly controlled and the deubiquitinases (DUBs) CYLD and OTULIN cleave M1-linked polyUb with high specificity and efficiency (Keusekotten et al., 2013; Komander et al., 2009b; Rivkin et al., 2013). Indeed, OTULIN and CYLD bind to LUBAC via a PUB (peptide:N-glycanase/UBA- or UBX-containing proteins) domain in HOIP. While OTULIN binds HOIP directly through its PUB-interacting motif (PIM) (Elliott et al., 2014; Schaeffer et al., 2014), CYLD is recruited via a PIM in the bridging factor SPATA2 (Elliott et al., 2016; Kupka et al., 2016; Schlicher et al., 2016; Wagner et al., 2016). PUB-PIM interactions with HOIP are mutually exclusive, and while OTULIN-LUBAC and CYLD/SPATA2-LUBAC complexes are readily detectable, only CYLD/SPATA2-LUBAC is efficiently recruited to TNFR complexes after TNF stimulation (Elliott et al., 2016; Kupka et al., 2016; Schlicher et al., 2016; Wagner et al., 2016). Nevertheless, OTULIN is also a negative regulator of TNF-α signaling (Damgaard et al., 2016; Hrdinka et al., 2016; Keusekotten et al., 2013). Furthermore, it serves to prevent auto-ubiquitination of LUBAC components at steady state, and accumulation of M1-linked chains in general (Elliott et al., 2014; Fiil et al., 2013; Keusekotten et al., 2013; Schaeffer et al., 2014). Genetically, an OTULIN loss-of-function mouse model displayed developmental defects in angiogenesis, leading to embryonic lethality (Rivkin et al., 2013). Conditional knockout (KO) mice with OTULIN deletion in immune cells demonstrated that OTULIN is critical for preventing spontaneous inflammation and maintaining immune homeostasis (Damgaard et al., 2016). This correlated with hypomorphic mutations in human patients and led to the description of an OTULIN-related auto-inflammatory syndrome (Damgaard et al., 2016; Zhou et al., 2016). Despite its pathophysiological importance, questions remain regarding whether and how the DUB activity and LUBAC interaction of OTULIN are regulated in cells.

Activity-based probes (ABPs) are powerful tools to study enzyme activities *in vitro* and *in vivo* and have been helpful for studying the activity of DUBs (Ekkebus et al., 2014). Whereas many DUBs react with ABPs containing an electrophilic group


© 2017 MRC Laboratory of Molecular Biology. Published by Elsevier Ltd. This is an open access article under the CC BY license (http://creativecommons.org/licenses/by/4.0/).

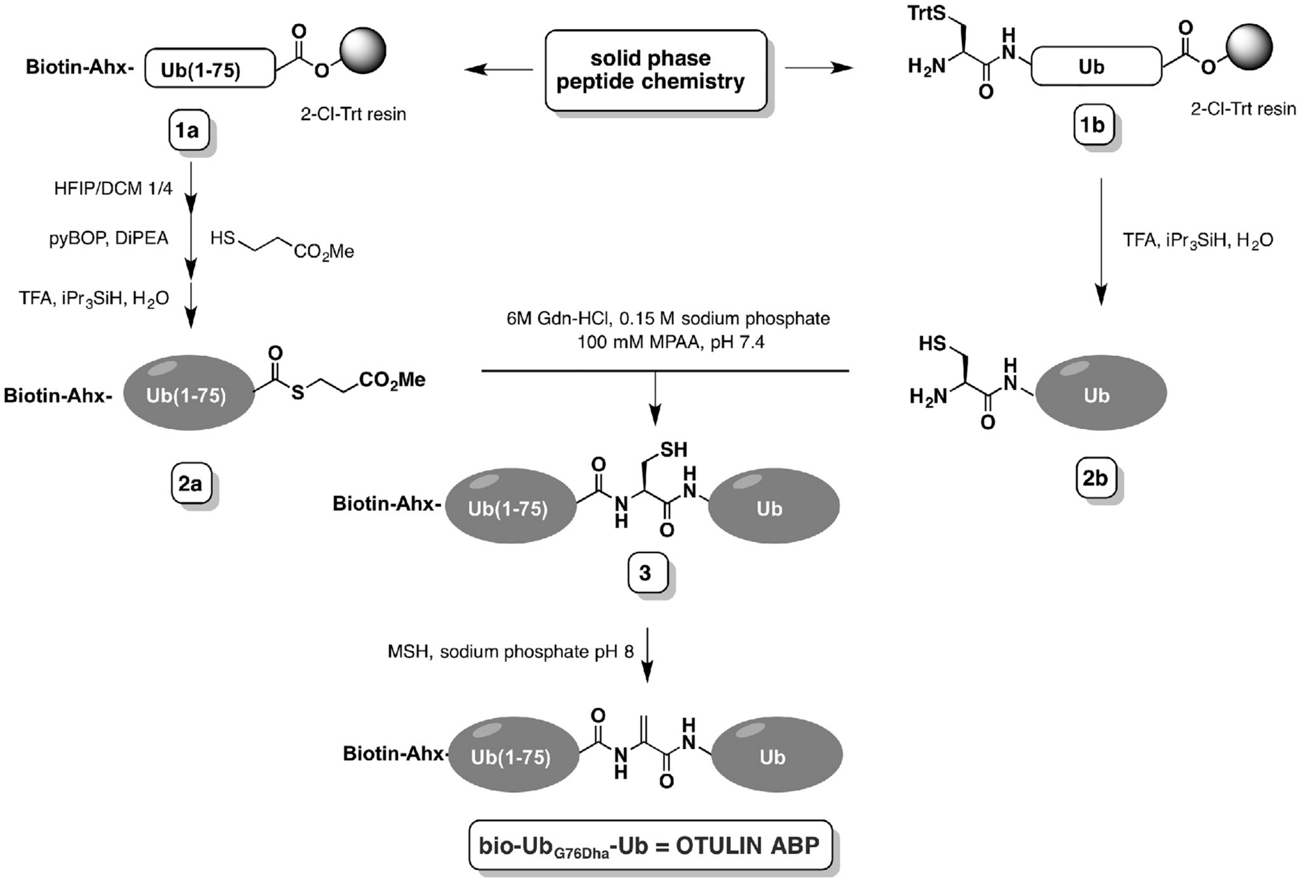


**Figure 1. Total Chemical Synthesis of Bio-Ub$_{G76Dha}$-Ub**

The synthesis is described in detail in the main text. See also Figure S1.

at the C terminus of a monoUb (de Jong et al., 2012), the substrate-assisted Ub chain hydrolysis of OTULIN relies on a native M1-linked diubiquitin (diUb) as a substrate (Keusekotten et al., 2013; Mevissen et al., 2013). The design of an electrophile that mimics the Gly-Met environment of the linear diUb linkage has remained a challenge to date. McGouran et al. (2013) designed an ABP based on an M1-linked diUb which more promiscuously labeled ubiquitin-specific protease (USP) DUBs that normally show little reactivity for linear Ub chains. OTULIN, however, was not labeled with this probe. The outcome was attributed to the greater flexibility of the used electrophile and the loss of key residues such as the M1 side chain and the amide bond between M1 and Gln2 of the proximal Ub (Keusekotten et al., 2013; McGouran et al., 2013). Mulder et al. (2014) reported the use of a chemical ligation handle that allowed the generation of linkage-specific diUb ABPs based on all seven lysine- and thus isopeptide-linked diUb chains (i.e., K6, K11, K27, K29, K33, K48, and K63). Whereas the design of the electrophile was well-suited to mimic the isopeptide bond between two Ub proteins, the differences in chemistry imposed by the "linear" peptide linkage made this strategy impractical for the M1-linked chain type. In another attempt to create an ABP based on linear diUb, M1 of proximal Ub was replaced by the electrophilic dehydroalanine (Dha) residue (Bernardes et al., 2008; Haj-Yahya et al., 2014). However, the probe was cleaved by OTULIN and USP2 rather than reacting covalently with the active site cysteine residues.

We report here on the total chemical synthesis of biotinylated linear diUb in which Gly76 of the distal Ub is replaced by Dha (bio-Ub$_{G76Dha}$-Ub: OTULIN ABP) to yield an ABP that covalently labels active OTULIN. By crystallizing the OTULIN-(Ub$_{G76Dha}$-Ub) complex, we show that the probe captures OTULIN in its active state. Proteome-wide mass spectrometry (MS) experiments reveal that the OTULIN ABP cross-reacts only with USP5 (Isopeptidase T) and the Ub-activating E1 enzymes UBA1 and UBA6. Deletion of the C-terminal glycine in the proximal Ub moiety in Ub$_{G76Dha}$-Ub$_{\Delta G76}$ abolishes USP5 labeling and E1 association, yielding a specific probe for OTULIN (OTULIN ABPΔG76). We show that the probes can be used to monitor cellular OTULIN activity, pull-down (PD) active OTULIN, and co-precipitate the OTULIN-associated E3 ligase LUBAC from cells.

## RESULTS

### Synthesis of Bio-Ub$_{G76Dha}$-Ub (OTULIN ABP)

Our strategy of generating a biotinylated Ub$_{G76Dha}$-Ub probe (Figure 1) is based on the earlier reported concept of using a 2-aminothiol residue (in this case Cys) to ligate a proximal and

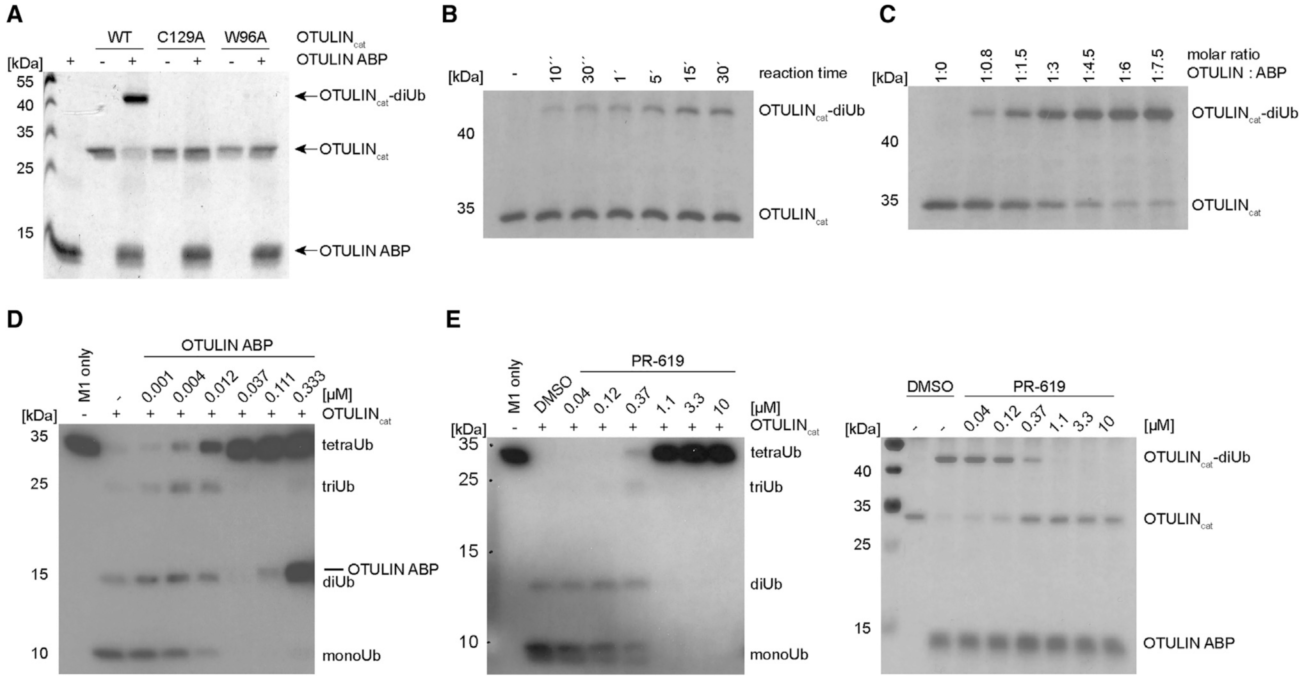


**Figure 2. Recombinant OTULIN Is Labeled by the OTULIN ABP**

(A) Recombinant $OTULIN_{cat}$ (1 μg) was incubated with 4 μg bio-$Ub_{G76Dha}$-Ub (2 hr, 37°C). OTULIN-diUb complex formation was analyzed by Coomassie staining.

(B) $OTULIN_{cat}$ (250 ng) was incubated at 30°C in a 1:1 molar ratio with OTULIN ABP. OTULIN-diUb complex formation was monitored by Silver staining.

(C) $OTULIN_{cat}$ (250 ng) was incubated with increasing OTULIN ABP concentrations (15 min, 30°C). OTULIN-diUb complex formation was monitored by Silver staining.

(D) TetraUb cleavage assay was performed after pre-treatment of OTULIN with increasing ABP concentrations. TetraUb chain cleavage was visualized by western blot.

(E) Left: TetraUb cleavage assay was performed as in (D) in the presence of increasing PR-619 concentrations. Right: $OTULIN_{cat}$ (500 ng) was pre-treated with PR-619 before incubation with 2 μg OTULIN ABP (5 min, 30°C). OTULIN-diUb complex formation was analyzed by Silver staining.

distal Ub part followed by its oxidative elimination to form an electrophilic residue (in our case Dha) (Mulder et al., 2014). Using the previously reported linear Fmoc-based solid-phase peptide synthesis of the Ub polypeptide (El Oualid et al., 2010), we synthesized biotin-labeled Ub(1–75) (**1a**). Here an aminohexanoic acid linker was introduced to create extra space between the biotin label and N terminus of Ub, allowing efficient access of biotin-binding entities (de Jong et al., 2012). The biotin-labeled Ub(1–75) was selectively cleaved from the 2-chlorotrityl resin using 20% 1,1,1,3,3,3-hexafluoroisopropanol in DCM, allowing for pyBOP-mediated coupling of methyl 3-mercaptopropionate at the C terminus. Global deprotection with 90% trifluoroacetic acid and purification by high-performance liquid chromatography (HPLC) gave the desired biotin-labeled Ub(1–75) thioester (**2a**) in 42% overall yield on a 40 μmol scale. Next, Cys-Ub (**2b**), representing the proximal part of our diUb probe design, was synthesized in a similar fashion and displayed good overall yield of 20% on a 25 μmol scale. Native chemical ligation of **2a** (30 mg/mL) and **2b** (26 mg/mL) was performed in 6 M Gdn-HCl, 0.15 M sodium phosphate, pH 7.4, with mercaptophenylacetic acid (MPAA, 100 mM) as ligation catalyst (El Oualid et al., 2010). Liquid chromatography-mass spectrometry (LC-MS) analysis showed that overnight incubation at 37°C resulted in full consumption of the proximal Ub mutant **2b** and formation of the ligation product as MPAA disulfide. A short treatment with Tris(2-carboxyethy1)phosphine followed by preparative HPLC gave the diUb conjugate (**3**) in 57% yield. Finally, the Cys residue was completely transformed into the electrophilic Dha, by treating a 2 mg/mL solution of **3** (30 mg) in 100 mM sodium phosphate, pH 8, overnight at 37°C with 15 eq of *O*-mesitylenesulfonylhydroxylamine (MSH). The desired probe bio-$Ub_{G76Dha}$-Ub (OTULIN ABP) (Figure 1) was isolated in 71% yield (21 mg, 1.2 μmol) after HPLC purification (Figure S1).

## OTULIN ABP Covalently Attaches to the Active Site of OTULIN

We incubated bio-$Ub_{G76Dha}$-Ub with the catalytic domain of OTULIN ($OTULIN_{cat}$, residues 80–352), which is sufficient for specific binding and cleavage of M1-linked Ub chains by OTULIN (Keusekotten et al., 2013). The probe was covalently attached to $OTULIN_{cat}$ as evident from a molecular-weight shift in an SDS-PAGE that corresponds to the expected 17 kDa shift in migration (Figure 2A). The probe labeled only active OTULIN, because C129A substitution in the catalytic center completely abolished covalent attachment of the probe. Thus, bio-$Ub_{G76Dha}$-Ub represents the first ABP that couples to the active site of OTULIN (OTULIN ABP). In addition, a point mutation in the S1′ site of OTULIN, W96A, previously shown to severely reduce affinity for M1-linked diUb (Keusekotten et al., 2013), also abrogated the assembly of the $OTULIN_{cat}$-diUb

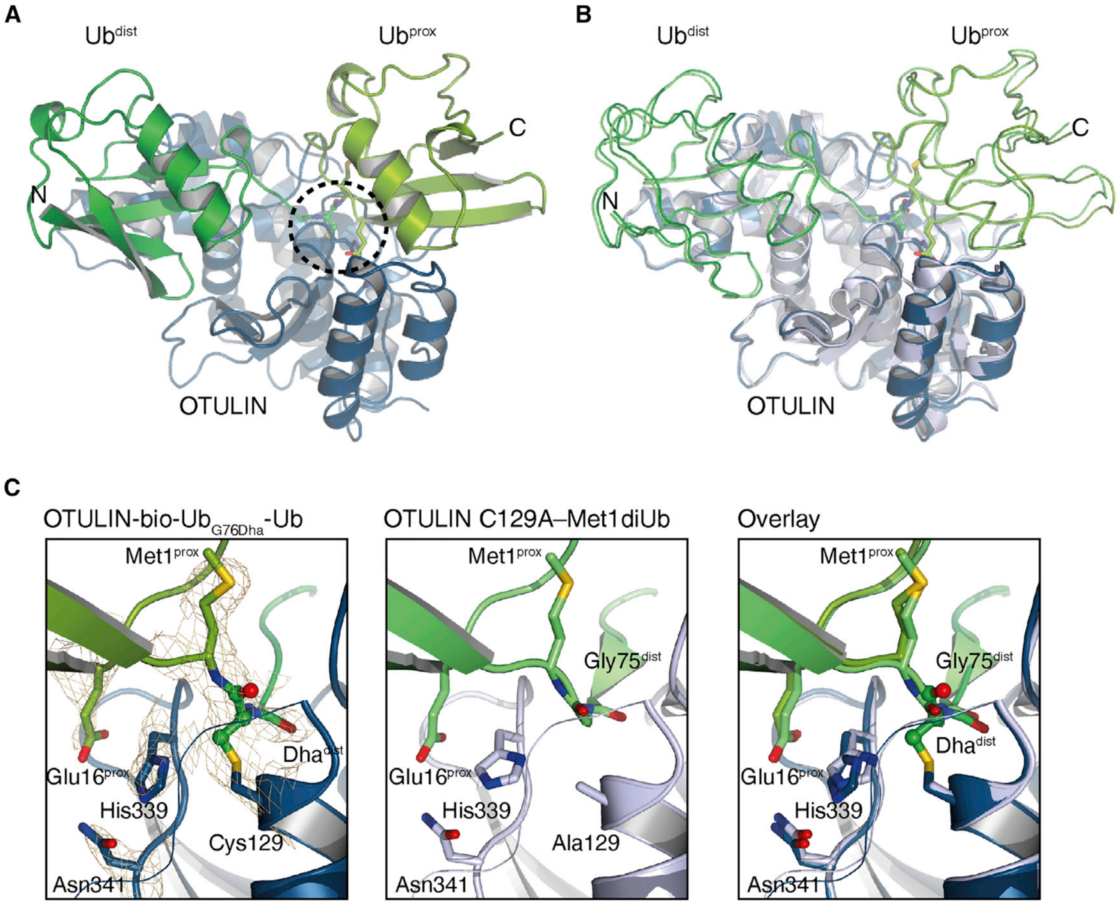


**Figure 3. Structural Analysis of the OTULIN-bio-Ub$_{G76Dha}$-Ub Complex**

(A) Structure of the covalent OTULIN$_{cat}$-bio-Ub$_{G76Dha}$-Ub complex. OTULIN (amino acids [aa] 80–352) shown in blue with the distal and proximal ubiquitin moieties of OTULIN ABP colored in dark and light green, respectively. Circle: site of the OTULIN catalytic triad. See also Table S1.

(B) Superimposition of the OTULIN catalytic domains from the OTULIN-bio-Ub$_{G76Dha}$-Ub structure (dark blue) with the OTULIN C129A M1-linked diUb complex (OTULIN C129A; light blue) (PDB: 3ZNZ). The diUb moieties are shown as ribbon to highlight the near identical arrangement.

(C) Close-up views of the OTULIN-bio-Ub$_{G76Dha}$-Ub active site (left) and the OTULIN C129A M1-diUb complex active site (middle). Residues 118–128 are shown as ribbons. Catalytic residues are shown in stick format, as is the Glu16prox residue. The Dha group is shown additionally as spheres. A simulated annealing composite omit map, contoured at 1.2$\sigma$ is shown encompassing the key catalytic residues and the Dha group. Right: superimposition of the two structures highlighting the identical arrangement of the catalytic triad.

product (Figure 2A). To analyze kinetics of the reaction between the OTULIN ABP and OTULIN, both proteins were incubated at 30°C and equimolar concentrations. The OTULIN$_{cat}$-diUb product was visible within 10–60 s, but we did not observe full conversion into the product within 30 min (Figure 2B). An approximately 5-fold molar excess of OTULIN ABP was necessary for an almost complete coupling of recombinant OTULIN$_{cat}$ within 15 min (Figure 2C). As expected for a covalent modifier, the irreversibly reacting ABP efficiently inhibited cleavage of linear tetraUb chains by OTULIN$_{cat}$ in a dose-dependent manner (Figure 2D). Under these *in vitro* conditions, OTULIN ABP prevented cleavage at concentrations between 12 and 37 nM. To confirm that OTULIN activity is needed for coupling to OTULIN ABP, we used the DUB inhibitor PR-619, which besides OTULIN also inhibits a wide range of DUBs, just as de-neddylating and desumoylating enzymes (Altun et al., 2011; Chen et al., 2014). Indeed, PR-619 blocked cleavage of M1-linked tetraUb chains by OTULIN, as well as the formation of the OTULIN-diUb product, in a dose-dependent manner and at similar concentrations (Figure 2E). These data demonstrate that the OTULIN ABP efficiently labels the active site of recombinant OTULIN$_{cat}$.

To verify that the ABP is able to capture OTULIN through its catalytic cysteine, we crystallized the OTULIN-bio-Ub$_{G76Dha}$-Ub complex. The structure was determined from crystals grown in similar conditions as the earlier reported OTULIN C129A-M1diUb complex (Keusekotten et al., 2013) and belonged to the same space group with near identical cell dimensions (Table S1) and low root-mean-square deviation (0.51 Å for OTULIN catalytic domain and 0.55 Å for diUb) (Figures 3A and 3B). Although the resolution of the OTULIN-bio-Ub$_{G76Dha}$-Ub complex was lower than the non-covalent OTULIN C129A-M1diUb structure (3.0 versus 1.9 Å, respectively), the electron density in the well-ordered active site enabled complete building of the thioether bond formed by the reaction between the Dha and C129 of OTULIN (Figure 3C). The obtained structure deviates only minimally from the previous non-covalent complex structure

(PDB: 3ZNZ; Keusekotten et al., 2013): the position and interactions of the distal and proximal Ub molecules are identical, and the mechanism of Ub-assisted catalysis provided by the proximal Ub molecule in the M1-linked chain, enabled the probe to react with the activated Cys.

## OTULIN ABP Selectively Labels the DUBs OTULIN and USP5

To evaluate the selectivity of OTULIN ABP for OTULIN, we first tested its reactivity *in vitro* against a panel of DUBs from the OTU (ovarian tumor), USP, and UCH (ubiquitin C-terminal hydrolases) families (Komander et al., 2009a). Whereas OTULIN ABP could quantitatively label OTULIN, we did not observe any reactivity with the K48-selective DUBs, A20 and OTUB1, which is putatively the closest homolog within the OTU family (Figure 4A) (Mevissen et al., 2013). Neither UCHL1 nor UCHL3 were modified by the ABP, which is in line with structural constraints that do not allow efficient cleavage of Ub polymers (Komander et al., 2009a). Also, several USPs (USP2, USP7, and USP8) did not react with the probe, although cleavage by many USPs is not restricted to Ub chain topology. Only a fraction of USP5 (Isopeptidase T) reacted with the OTULIN ABP. Since OTULIN and USP5 are able to cleave M1-linked polyUb, we also tested *in vitro* reactivity with the other M1-hydrolyzing DUBs USP2, USP21, and CYLD (Komander et al., 2009b; Ye et al., 2011). However, OTULIN ABP did not react with these USP DUBs, revealing that, among M1-hydrolyzing DUBs, the probe displays a high selectivity for OTULIN (Figures 4A and 4B). All DUBs were active, which was verified either by coupling to the monoUb-based ABP ubiquitin-propargylamide (Ub-PA) (Ekkebus et al., 2013) or by cleavage of K48-linked diUb (Figures S2A and S2B). Given the M1- and K63-selective cleavage of CYLD, the lacking coupling to OTULIN ABP was unexpected. Thus, we directly compared labeling of OTULIN and CYLD (583–956) with OTULIN ABP and Ub-PA at equimolar concentrations and again found no coupling of OTULIN ABP to CYLD (Figure S2C). Indeed, when comparing the structures of OTULIN-Ub$_{G76Dha}$-Ub and non-covalent zebrafish CYLD (C596S) Met1-diUb complexes (Sato et al., 2015), it becomes clear that CYLD has a narrow channel that binds the distal Ub tail (Figures 4C and 4D). There is limited space in the CYLD active site around the Gly76-Met1 peptide bond, as CYLD Asp794, Lys756, and Ser800 form a tight channel. Most likely, this prevents OTULIN ABP from reaching the catalytic Cys in CYLD.

Interestingly, the G76Dha substitution in the distal Ub of the probe did not prevent covalent binding to OTULIN, despite the fact that G76S substitution in an M1-linked tetraUb chain abolished recognition and cleavage by OTULIN (Keusekotten et al., 2013). Also, it has been reported that the USP domain-containing DUB CYLD requires Gly76 in the distal Ub for proper substrate recognition and cleavage (Komander et al., 2008; Sato et al., 2015). In line with our probe design, we wanted to determine whether the G76A substitution in diUb (Ub(1–75)-Ala-Ub: Ub$_{G76A}$-Ub) would be tolerated. OTULIN, USP21, and CYLD were unable to cleave the G76A mutant of M1-linked diUb (Figure 4E). Next, we tested the involvement of the distal Ub G76A in OTULIN binding. For this, we performed PDs of recombinant Strep-tag II-tagged OTULIN$_{cat}$-(ST) together with either linear diUb or Ub$_{G76A}$-Ub (Figure 4F). As expected, linear diUb was cleaved by wild-type (WT) OTULIN$_{cat}$, and pulled down by the catalytically inactive OTULIN$_{cat}$ C129A mutant. In contrast, Ub$_{G76A}$-Ub was not cleaved by OTULIN$_{cat}$(-ST), and also binding to OTULIN WT or OTULIN C129A was severely diminished (Figure 4F). Thus, Gly76 in the distal Ub contributes to the non-covalent association of OTULIN to M1-linked diUb. However, there is still sufficient binding to enable a covalent reaction of OTULIN with the Dha electrophile in our probe.

## OTULIN ABP Selectively Labels OTULIN and USP5 in Cell Extracts

Next, we examined if the OTULIN ABP could also react with OTULIN in cell lysates. We expressed Flag-OTULIN WT and C129A in HEK293 cells and incubated the cell lysates after extraction directly with the probe. OTULIN WT but not the C129A mutant was quantitatively labeled by the probe, as demonstrated by western blot analysis with anti-Flag antibody (Figure 5A). Further probing with an OTULIN antibody indicated that endogenous OTULIN also completely reacted with the probe. Again, we confirmed the specificity toward OTULIN by monitoring cross-reactivity with several DUBs after overexpression and at endogenous levels (Figures S3A and 5B). We tested OTUB1, YOD1, A20 (all OTU domain), CYLD and USP5 (USP domain), and UCHL3 (UCH domain). With the exception of USP5, which was partially labeled by the OTULIN ABP, none of the other DUBs reacted with the probe. We also determined efficacy of the OTULIN ABP reaction with cellular OTULIN and USP5 in titration and kinetic experiments (Figures 5C and 5D). At room temperature, 1 $\mu$g of OTULIN ABP was quantitatively labeling endogenous OTULIN in extracts from 2 $\times$ $10^7$ Jurkat T cells within 15 min. Similar to the *in vitro* experiments (Figure 2), an excess of OTULIN ABP is required for efficient labeling, but the slower and partial reactivity with USP5 underscores the high preference of the probe for coupling to OTULIN. Thus, the data provide evidence that, within the analyzed panel of DUBs, the OTULIN ABP labels OTULIN with high selectivity and only partially cross-reacts with USP5.

To obtain a better picture of the selectivity of the OTULIN ABP, we incubated extracts of HEK293 cells with OTULIN ABP and performed western blots with a specific anti-M1-polyUb antibody that also reacts with the linear diUb-based probe and thus recognizes labeled proteins. Indeed, the OTULIN-diUb product was clearly detectable with the anti-M1-polyUb antibody, but prolonged incubation times for 30 min at 30°C in the extracts led to the appearance of a high-molecular-weight smear that was reminiscent to M1-linked polyUb chains (Figure S3B). We depleted ATP from the extracts by apyrase treatment. Clearly, the *in vitro* polyubiquitination that was detected with anti-M1-polyUb and anti-Ub antibodies relies on an active ATP-dependent process, suggesting that assembly of polyUb smear results from an enzymatic ubiquitination reaction (Figure 5E). Two prominent bands were detected by the anti-M1-polyUb antibody at ~60 and ~130 kDa upon OTULIN ABP incubation, and anti-OTULIN and anti-USP5 antibodies confirmed that these bands correspond in migration to the OTULIN-diUb and USP5-diUb products (Figure 5E). Importantly, despite some weaker background signals, OTULIN and USP5 were the major OTULIN ABP adducts detected with the

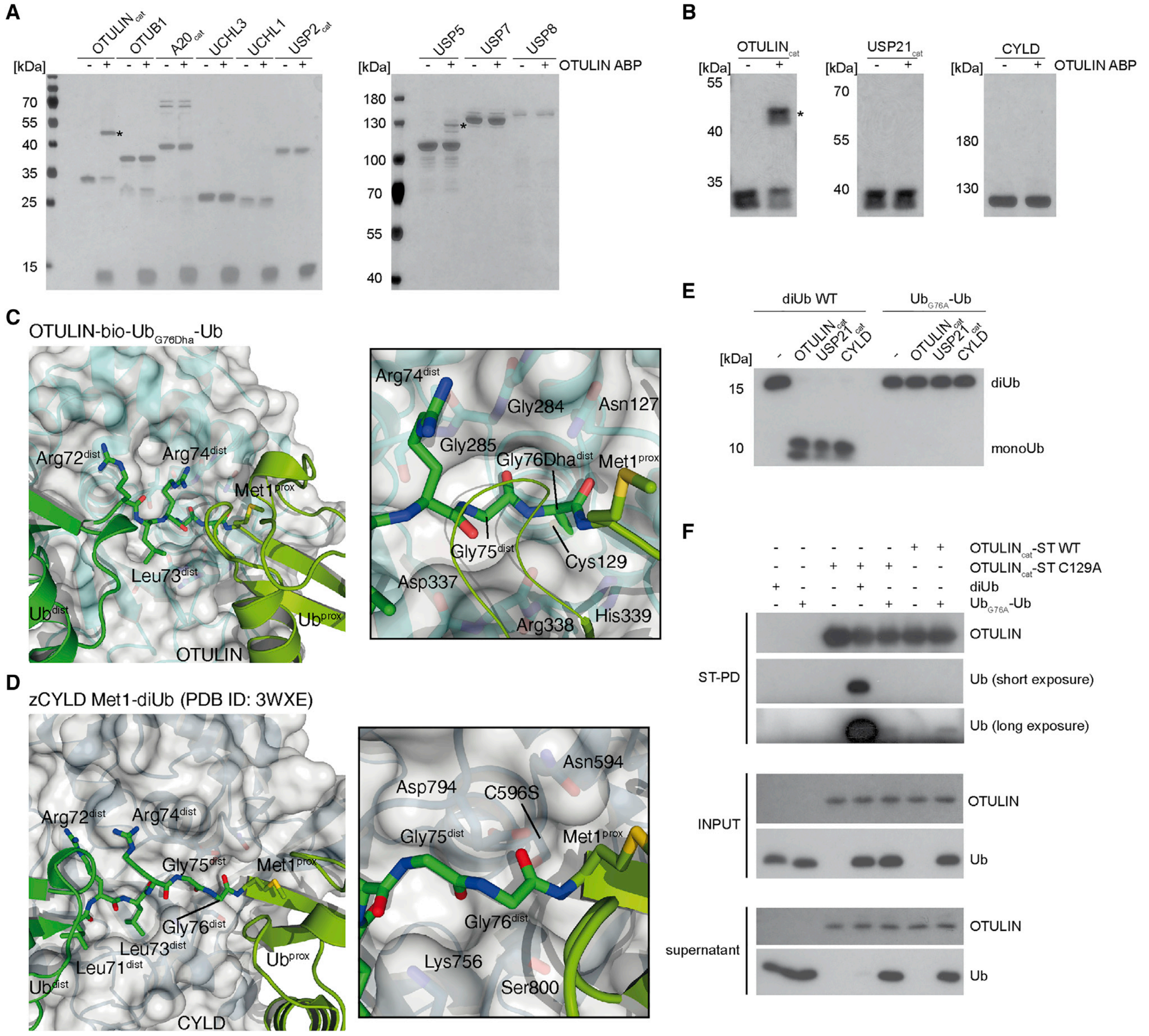


**Figure 4. OTULIN ABP Shows Minimal Cross-Reactivity with Other Recombinant DUBs**

(A and B) DUBs (0.5–1 μM) were incubated with 3 μM OTULIN ABP (1 hr, 30°C). Labeling was analyzed by Silver staining. Asterisks: DUB-diUb adducts. See also Figures S2A and S2B.

(C) Close-up of the structure of the catalytic site of $OTULIN_{cat}$ (aa 80–352; white surface) reacted to the bio-$Ub_{G76Dha}$-Ub probe (green cartoon). Insert, OTULIN residues that form the substrate channel are shown in stick representation.

(D) Close-up of structure of the catalytic site of zebrafish CYLD (zCYLD) (aa 578–951, C598S; white surface) bound to Met1-linked diUb (green cartoon) (PDB: 3WXE). Insert, residues from CYLD that form a tight substrate channel around the Gly76-Met1 peptide bond are shown in stick representation.

(E) Recombinant $OTULIN_{cat}$, $USP21_{cat}$, or CYLD (1 μg) were incubated with either M1-linked diUb WT or $Ub_{G76A}$-Ub (1 hr, 37°C). diUb cleavage was analyzed by western blot.

(F) ST-$OTULIN_{cat}$ (WT or C129A) was pulled down with Strep-Tactin beads in the presence of M1-linked diUb or $Ub_{G76A}$-Ub. Interaction between diUb and $OTULIN_{cat}$ proteins was analyzed by western blot.

anti-M1-polyUb antibody after ATP depletion, suggesting that the probe is coupling quite selectively to these two enzymes.

Next, we assessed OTULIN ABP specificity in cellular extracts on a global scale by MS. To this end, we performed biotin-PDs to enrich bio-$Ub_{G76Dha}$-Ub binders. To select for covalent interactors, we optimized biotin-PD stringency. Up to 1% SDS in the washing buffer did not significantly reduce detection of OTULIN-diUb complexes, but strongly diminished the known non-covalent interaction to HOIP (Figure S4A; see also below). We prepared four biological replicates for each sample depicted in Figure 6A. Besides comparing biotin-PD with and without OTULIN ABP (samples 1 and 2), we also depleted ATP by

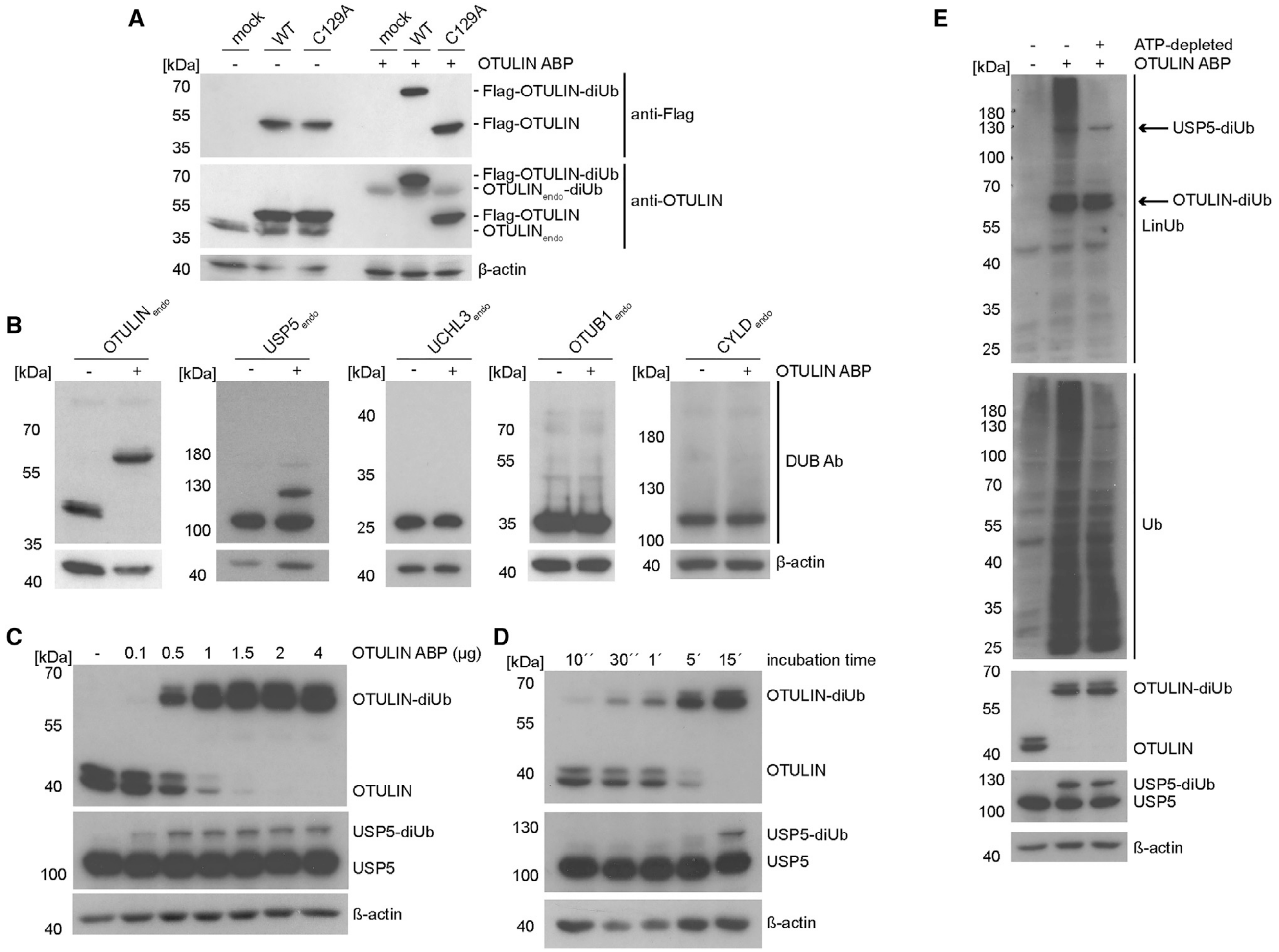


**Figure 5. In Cell Extracts, OTULIN ABP Labels OTULIN and USP5, and Induces PolyUb Chain Formation**

(A) Extracts of Flag-OTULIN WT or C129A overexpressing HEK293 cells ($\sim 2.5 \times 10^5$ cells/reaction) were incubated with 1 μg OTULIN ABP (30 min, 30°C). Labeling of exo- and endogenous OTULIN was analyzed by western blot.

(B) Cross-reactivity of OTULIN ABP with endogenous DUBs. Incubation of OTULIN ABP in HEK293 extracts was performed as in (A). See also Figure S3A.

(C and D) Extracts of Jurkat T cells ($2 \times 10^7$ cells) were incubated at room temperature with increasing OTULIN ABP amounts for 15 min (C) or with 1 μg OTULIN ABP for different times (D).

(E) For ATP depletion, extracts from $6 \times 10^5$ HEK293 cells were incubated with apyrase prior to the incubation with 1 μg OTULIN ABP for 30 min. Ubiquitin chain formation and ABP adducts were analyzed by western blot. See also Figure S3B.

apyrase treatment to inhibit interactions that may arise from the conjugation of the probe into polyUb chains (sample 3). In addition, we checked for covalent coupling by adding the broad-spectrum DUB inhibitor PR-619 (Altun et al., 2011; Chen et al., 2014) that also prevents labeling of OTULIN by OTULIN ABP (see Figure 1E; sample 4). As expected, whereas PR-619 abolished coupling and pull-down of OTULIN-diUb, apyrase treatment did not have an affect (Figure S4B).

We first searched for all DUBs that were identified in the samples by liquid chromatography-tandem mass spectrometry (LC-MS/MS) (Table S2). Only three DUBs were identified and the statistical comparison of sample 1 (no probe) and sample 2 (OTULIN ABP) showed that OTULIN, USP5, and UCHL3 were significantly enriched in the OTULIN ABP-bound sample (volcano plot, Figure 6B). Moreover, treatment with DUB inhibitor PR-619 led to the significant depletion of only one single DUB, OTULIN (Figure 6C). These results suggest that despite the partial labeling of USP5 in cell extracts, USP5 and UCHL3 are primarily bound to the OTULIN ABP in a non-covalent and reversible manner. To confirm this result, we performed PD experiments treating the samples analogous to the MS experiment but using less stringent washing conditions after PD (Figure 6D). As previously observed, the OTULIN ABP labeled OTULIN and USP5, but not UCHL3, in the extracts (Figure S4C). Indeed, whereas OTULIN was exclusively bound in the modified form of OTULIN-diUb, USP5 and UCHL3 were detected only in the unmodified form, revealing that these DUBs have a high affinity for the linear diUb probe, but are either not at all or just poorly coupled (Figure 6D). Whereas previous assays showed that UCHL3 does not attack the probe, the OTULIN ABP directly labels USP5 when lysates are incubated with ABP (see Figure 5B). Thus, combined activity profiling by MS and western blotting

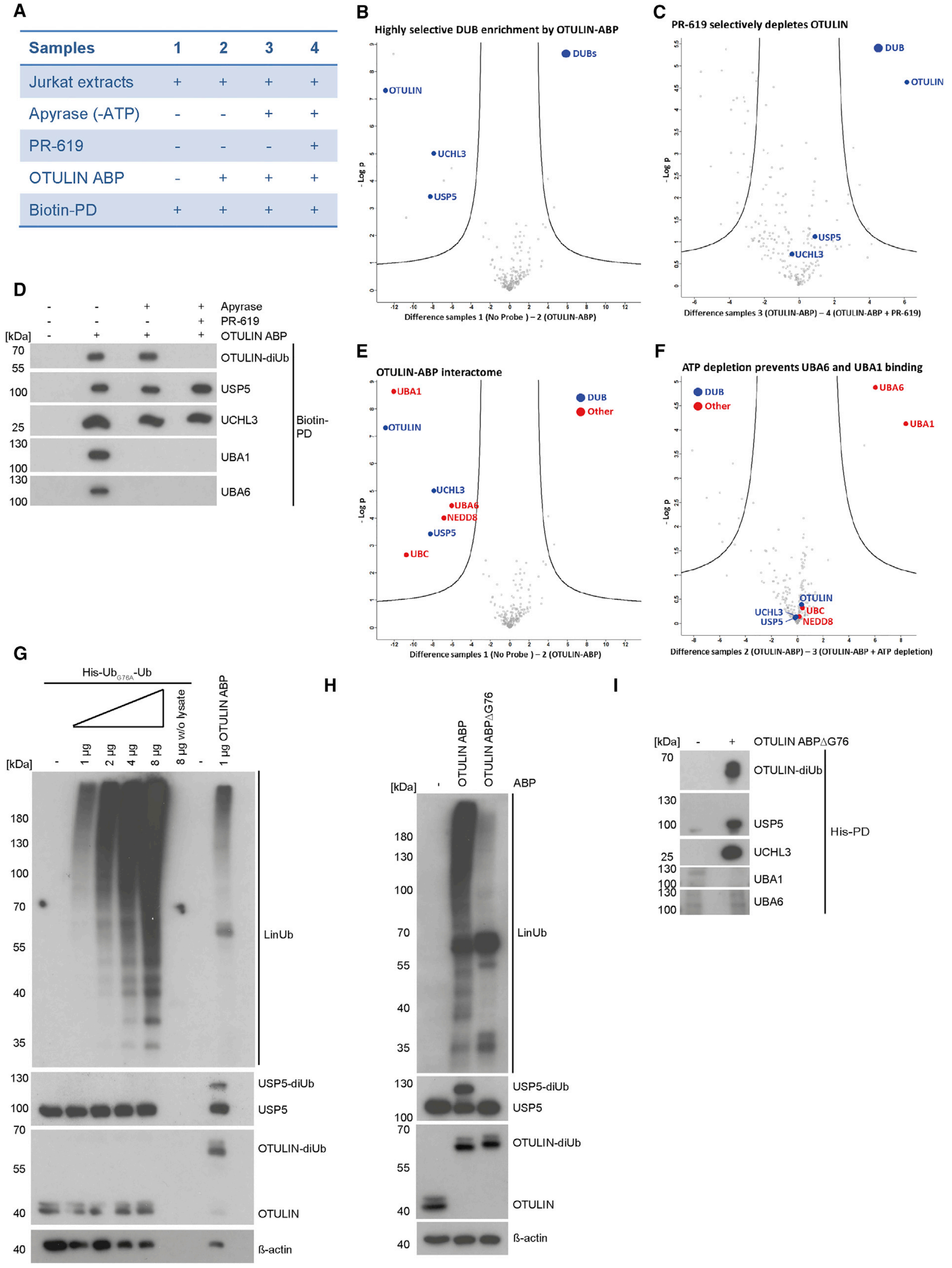


*(legend on next page)*

proves that the OTULIN ABP shows high selectivity for OTULIN with some cross-reactivity toward USP5.

Moreover, when we determined global reactivity of OTULIN ABP by comparing the whole interactome enriched by biotin-PD in sample 2 versus sample 1 (Table S2 and Figure 6E), only four other proteins (red) bound to OTULIN ABP besides the three DUBs (blue). These include the Ub precursor UBC, which most likely reflects detection of the probe itself, and the Ub-like modifier NEDD8. In light of the ATP-dependent Ub chain assembly in the OTULIN ABP-treated extracts it was very remarkable that the two mammalian Ub-activating enzymes, UBA1 and UBA6, were bound to the OTULIN ABP. In addition, UBA1 and UBA6 were the only associated proteins whose binding was lost after ATP depletion (Figures 6E and 6F), which was confirmed by western blotting after biotin-PD (Figure 6D). Interaction to all of these proteins was resistant to PR-619 treatment, indicating that they are not associating to the OTULIN ABP via OTULIN (Figure S4D).

The ATP-dependency suggests that the C-terminal carboxyl group of the diUb probe can form a thioester bond with the E1s, just like in the first monoUb activation step in a ubiquitination cascade (Chen and Pickart, 1990; Martinez-Fonts and Matouschek, 2016). Since thioesters are labile under reducing conditions, no molecular-weight shift was detected for UBA1 and UBA6 by SDS-PAGE (Figure S4C). Nevertheless, the MS data indicate that the ATP-dependent assembly of polyUb chains by the probe may not be based on inhibition of OTULIN or other DUBs. The data instead suggest that the OTULIN ABP itself is activated by E1s and conjugated to form polyUb chains. Therefore, we determined if the non-inhibitory, but cleavage-resistant, diUb protein His-$Ub_{G76A}$-Ub (see Figure 4E) is also able to form polyUb chains (Figure 6G). As expected, $Ub_{G76A}$-Ub did not label or inhibit OTULIN and USP5, but it also induced the dose-dependent assembly of Ub chains that could be detected with anti-M1-polyUb antibody (Figure 6G). The assumption that uncleavable diUbs (such as the OTULIN ABP) are incorporated into unanchored chains was further supported by biotin-PDs, which not only precipitated and depleted free bio-$Ub_{G76Dha}$-Ub probe from the cell extracts, but also removed the formed polyUb smear (Figure S4E). In line, the polyUb chains were strongly detected with the M1-specific anti-M1-polyUb antibody that also reacts with the bio-$Ub_{G76Dha}$-Ub probe and His-$Ub_{G76A}$-Ub. Since the N-terminal Tags prevent a head-to-tail linkage of the diUb probes, we performed Ub chain linkage analyses using the UbiCREST technology (Figure S4F) (Hospenthal et al., 2015). Recombinant OTULIN was coupled and inhibited by OTULIN ABP (see asterisk), but even in excess it was not able to cleave the chains, supporting the notion that the chains are assemblies of bio-$Ub_{G76Dha}$-Ub adducts. Only the promiscuous DUB USP2, which cleaves all types of Ub linkages, was able to completely remove the high-molecular-weight Ub smear induced by OTULIN ABP. YOD1, which hydrolyzes primarily the atypical K6, K11, K27, K29, and K33 linkages, was able to partially reduce the Ub smear when used at high concentrations (Figure S4G). Thus, the data indicate that various lysine residues in the OTULIN ABP and His-$Ub_{G76A}$-Ub protein can be ubiquitinated.

## OTULIN ABPΔG76: A Specific OTULIN ABP

Besides serving as the site for thioester formation by E1 enzymes, the proximal C-terminal Gly76 residue in a Ub chain is essential for USP5 activity (Reyes-Turcu et al., 2006). As such, we reasoned that a diUb probe lacking this C-terminal glycine should not exhibit any cross-reactivity with UBA1/UBA6 and USP5. For the synthesis of this probe we combined bacterial protein expression with chemical synthesis (Figure S4H). A $His_{10}$-tagged diUb variant was cloned in which Gly76 of the proximal Ub was deleted and Gly76 of the distal Ub was replaced by cysteine ($His_{10}$-$Ub_{G76C}$-$Ub_{\Delta G76}$). After bacterial expression and purification, the Cys residue was completely transformed into Dha by treatment with MSH (as judged by LC-MS; Figure S4I) affording the desired $His_{10}$-$Ub_{G76Dha}$-$Ub_{\Delta G76}$ (OTULIN ABPΔG76).

When we compared the reactivity of OTULIN ABP (bio-$Ub_{G76Dha}$-Ub) and OTULIN ABPΔG76 ($His_{10}$-$Ub_{G76Dha}$-$Ub_{\Delta G76}$) in extracts of HEK293 cells, both probes converted OTULIN to the higher migrating OTULIN-diUb adduct (Figure 6H). Importantly, the OTULIN ABPΔG76 no longer showed any cross-reactivity with USP5 (Figure 6H). His-PD showed that OTULIN ABPΔG76 still bound to USP5 and UCHL3 (Figure 6I), confirming our MS results that these DUBs are associating largely in a non-covalent manner with the probes (compare Figures 6B and 6C). Gratifyingly, formation of Ub chains and binding to E1 enzymes were abolished in the case of OTULIN ABPΔG76 (Figures 6H and 6I), indicating that the probe can no longer be activated by UBA1/UBA6. Thus, by removing the C-terminal Gly76 in the

**Figure 6. LC-MS/MS Reveals Highly Selective OTULIN Coupling and E1-Dependent Auto-Conjugation of OTULIN ABP**

(A) Overview of ABP-PD samples analyzed by LC-MS/MS.

(B) Volcano plot demonstrating enrichment of identified DUBs (blue) after biotin-PD in the presence (sample 2) compared with the absence (sample 1) of OTULIN ABP. Curves depict significant enrichment or depletion, respectively. See also Table S2.

(C) Volcano plot demonstrating loss of DUB binding (blue) between control (sample 3) and PR-619 (sample 4) treatments before OTULIN ABP incubation and biotin-PD. Curves depict significant enrichment or depletion, respectively.

(D) Biotin-PDs from extracts of Jurkat T cells ($2 \times 10^7$) were performed using the same conditions as for LC-MS/MS analyses and analyzed by western blot.

(E) Volcano plot demonstrating selective binding of DUBs (blue) and other proteins (red) to OTULIN ABP (sample 1 versus 2). Curves depict significant enrichment or depletion, respectively.

(F) Volcano plot demonstrating loss of UBA1 and UBA6 binding to OTULIN ABP upon ATP depletion (sample 2 versus 3). Curves depict significant enrichment or depletion, respectively.

(G) Extracts of HEK293 cells ($6 \times 10^5$ cells) were incubated with increasing amounts of His-$Ub_{G76A}$-Ub or with 1 μg OTULIN ABP (45 min, 30°C). Ubiquitin chain formation and OTULIN-diUb or USP5-diUb complexes were analyzed by western blot using anti-M1-polyUb and DUB-specific antibodies.

(H) Extracts of HEK293 cells ($6 \times 10^5$ cells) were incubated with 1 μg OTULIN ABP or 1 μg OTULIN ABPΔG76 (45 min, 30°C). Labeling of OTULIN and USP5 as well as Ub chain formation was analyzed by western blot.

(I) Extracts of Jurkat T cells ($2 \times 10^7$ cells) were treated with OTULIN ABPΔG76 before His-PD. Interactions between the new probe and the indicated proteins were analyzed by western blot.

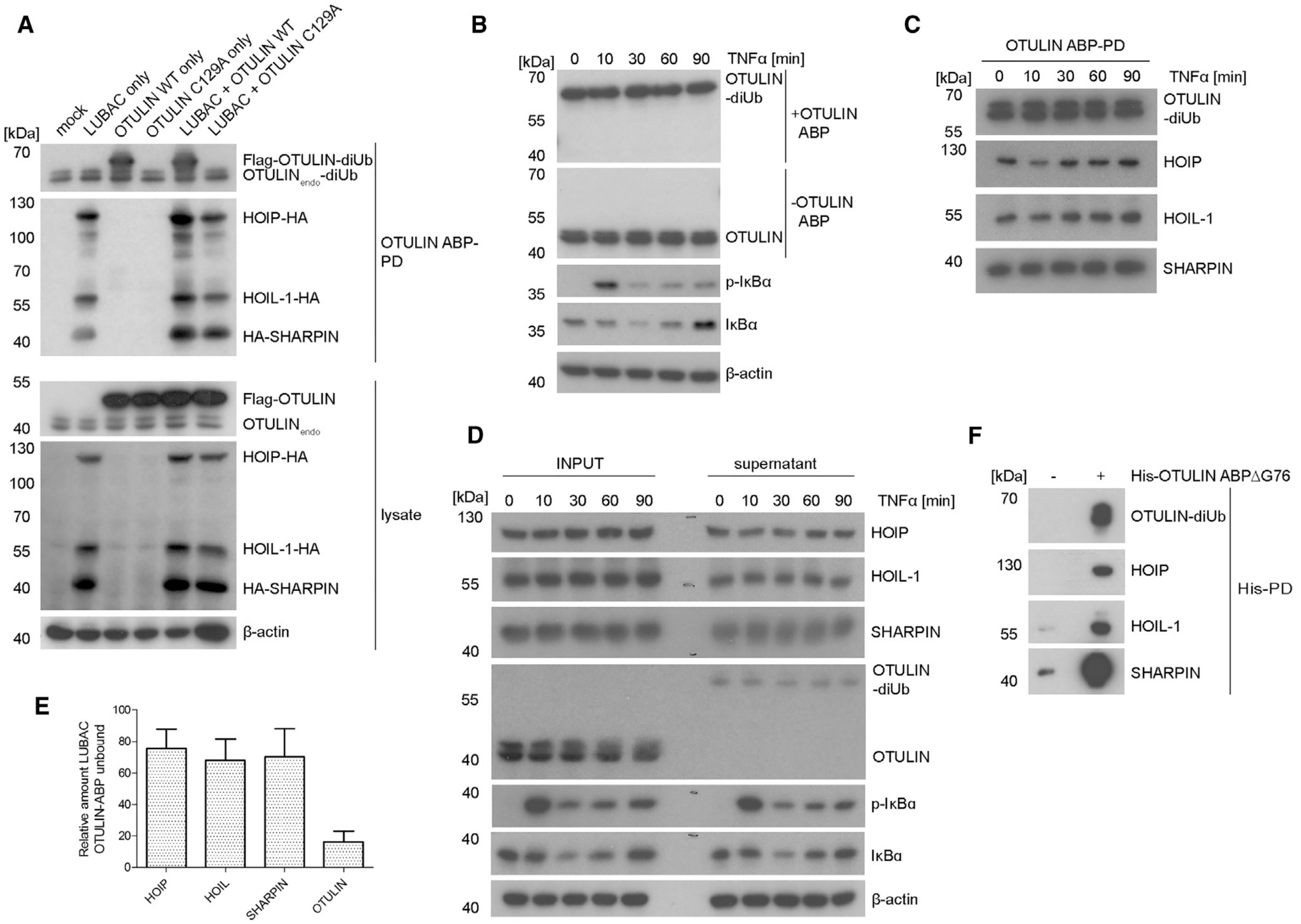


**Figure 7. Association of Substrate-Bound OTULIN with LUBAC**

(A) HA-tagged LUBAC components and Flag-tagged OTULIN were co-transfected into HEK293 cells as indicated. Biotin-PD of OTULIN-diUb after OTULIN ABP incubation in cell extracts (~1 × $10^7$ cells) was analyzed for the association of transfected LUBAC proteins by western blot.

(B) Jurkat T cells were stimulated with TNF-α (20 ng/mL), and lysates (2.5 × $10^5$ cells) were incubated with OTULIN ABP (1 μg) and analyzed for changes in OTULIN activity by western blot.

(C) Biotin-PD of OTULIN ABP was performed as in (A) from extracts of TNF-α-stimulated Jurkat T cells (2 × $10^7$ cells, 4 μg OTULIN ABP). Interaction of endogenous OTULIN-diUb and LUBAC was assessed by western blot.

(D) Cell extracts of Jurkat T cells (+/− TNF-α stimulation) were subjected to OTULIN ABP incubation. OTULIN, HOIP, HOIL-1, and SHARPIN amounts were determined by western blotting prior (input) and after (supernatant) biotin-PD of OTULIN-diUb complexes.

(E) The majority of LUBAC subunits is not associated with OTULIN-diUb complexes. Biotin-PD of OTULIN-diUb complexes from extracts of unstimulated Jurkat T cells (2 × $10^7$ cells) was performed as in (C). Levels of LUBAC subunits and OTULIN were quantified in the input and the supernatant. The quantified relative amounts of "free" OTULIN-diUb unbound proteins are depicted. Data represent the mean ± SD of six independent experiments.

(F) Extracts of Jurkat T cells (2 × $10^7$ cells/sample) were treated with 4 μg OTULIN ABPΔG76 before His-PD. Binding of LUBAC components after PD was analyzed by western blot.

proximal Ub moiety of our OTULIN ABP, the corresponding OTULIN ABPΔG76 becomes a specific OTULIN ABP.

## Substrate-Bound OTULIN Associates with LUBAC

The N-terminal PIM of OTULIN binds to the PUB domain of HOIP to control LUBAC activity (Elliott et al., 2014; Schaeffer et al., 2014). Size-exclusion chromatography demonstrated that recombinant OTULIN C129A, Met1-diUb, and HOIP (amino acids 1–182) can interact to form a ternary complex (Figure S5A). Thus, we wondered whether biotin-PD of OTULIN ABP can be used to monitor association of active substrate-bound OTULIN to HOIP, and potentially the entire LUBAC complex (Figure 7). For this, we expressed hemagglutinin (HA)-tagged HOIP, HOIL-1, and SHARPIN, together with Flag-OTULIN, in HEK293 cells (Figure 7A). Biotin-PD precipitated endogenous OTULIN as well as overexpressing Flag-OTULIN WT, but not catalytically inactive OTULIN C129A. HA-tagged HOIP, HOIL-1, and SHARPIN co-precipitated in similar amounts with the OTULIN-diUb adduct. Co-precipitation was slightly increased upon OTULIN overexpression, revealing that substrate-bound OTULIN is associated with LUBAC.

Generation of M1-linked Ub chains drives TNFR-induced NF-κB signaling (Sasaki and Iwai, 2015). Therefore, we determined whether OTULIN activity and OTULIN association with

LUBAC is altered upon TNF-α stimulation. Stimulation of HEK293 cells with TNF-α for 10 to 90 min led to NF-κB activation as evident by IκBα phosphorylation and degradation (Figure 7B). However, activity of OTULIN was not impaired upon TNF-α stimulation, as apparent from the unchanged formation of OTULIN-diUb complexes in extracts of TNF-α-treated HEK293 cells. To determine if stimulation alters the binding of endogenous OTULIN to LUBAC, we performed biotin-PD of OTULIN-diUb complexes from the extracts of Jurkat T cells prior to or after TNF-α treatment (Figure 7C). The three LUBAC components co-precipitated with substrate-bound OTULIN, but the amounts of HOIP, HOIL-1, or SHARPIN did not decrease after TNF-α stimulation (Figure 7C). To determine the amounts of LUBAC associated with the OTULIN ABP, we directly compared the levels of HOIP, HOIL-1, and SHARPIN by loading equal volumes of the lysates before (input) and after biotin-PD (supernatant) (Figure 7D). The PD reduced the amounts of LUBAC in the supernatant, but TNF-α stimulation did not affect association of substrate-bound OTULIN to LUBAC. To analyze more closely how much LUBAC is associated with substrate-coupled OTULIN, we quantified the concentration of HOIP, HOIL-1, and SHARPIN in the input (total LUBAC) and the supernatant ("free" OTULIN-diUb unbound LUBAC) in extracts from unstimulated Jurkat T cells (Figure 7E). Whereas the biotin-PD depleted more than 80% of active OTULIN, only approximately 25% of each LUBAC subunit were removed from the extracts by co-precipitation. This means that the majority of HOIP, HOIL-1, and SHARPIN (~75%) were not associated with substrate-bound OTULIN. *In vitro* experiments already indicated that the ternary HOIP-OTULIN-diUb complex is quite stable in gel filtration chromatography (Figure S5A). To make sure that the partial binding of LUBAC to substrate-bound OTULIN is not resulting from a high dissociation rate in PD experiments, we monitored the association in successive washing steps (Figure S5B). By increasing the number of washing steps, the detected amounts of OTULIN-diUb and bound LUBAC did not alter, indicating that the partial LUBAC binding to OTULIN-diUb is not a result of constant dissociation of the complex. Finally, to ascertain that the observed OTULIN-LUBAC association is independent of USP5 coupling and ATP-dependent conjugation of Ub chains by the probe, we verified that association of active OTULIN with the LUBAC was also detectable with the specific OTULIN ABPΔG76 (Figure 7F). The data indicate that separate LUBAC-associated complexes exist in cells that may be subject to differential regulation by different DUBs.

## DISCUSSION

With the synthesis of bio-$Ub_{G76Dha}$-Ub (OTULIN ABP) and His-$Ub_{G76Dha}$-$Ub_{\Delta G76}$ (OTULIN ABP ΔG76) we provide the first substrate-based diUb probes that covalently label the M1-specific DUB OTULIN. Together with the previously developed diUb ABP panel that comprises all linkage types (Mulder et al., 2014), the OTULIN ABP completes the toolkit of diUb-based DUB probes targeting the linkage specificity determining S1 and S1′ sites of an enzyme.

The design of the OTULIN probes is based on the assumption that, despite some rigid constraints for the binding of OTULIN to diUb, position Gly76 in the distal Ub moiety may be amendable for a nucleophilic attack by the catalytic Cys129 in OTULIN (Keusekotten et al., 2013). By determining the structure of OTULIN in complex with bio-$Ub_{G76Dha}$-Ub we confirmed that the electrophilic Dha76 covalently couples to OTULIN Cys129. In contrast, the OTULIN-diUb co-structures (PDB: 3ZNZ) clearly indicated that M1 of proximal Ub moiety is pointing away from the active site (Figure 3C), and, in line, the exchange of M1 to Dha failed to react with OTULIN (Haj-Yahya et al., 2014). Our structural analysis of the substrate bound to active WT OTULIN underscores the rigid constraints for OTULIN-diUb binding and thus the hydrolysis of M1-linked Ub leading to the high specificity of the enzyme. The mutation of G76A in the distal Ub moiety severely reduced the affinity to OTULIN and completely abrogated cleavage of diUb by OTULIN (Figures 4E and 4F) (Keusekotten et al., 2013). Indeed, there is very little space in the active site, and the alanine side chain at position Gly76 potentially clashes with catalytic Cys129 of OTULIN (Figures 3C and 4C). Nevertheless, despite the apparently lower affinity compared with the native linkage, the presence of the electrophile Dha is sufficient for being captured by the Cys129 of OTULIN. Thus, formation of the covalent OTULIN-diUb product is driven by the Dha electrophile and not so much by the affinity of OTULIN for the probe.

Most striking was the high selectivity of bio-$Ub_{G76Dha}$-Ub for the DUB OTULIN, as revealed by direct DUB assays *in vitro* as well as by binding studies using MS (Figures 4, 5, and 6). Surprisingly, the USP DUBs USP2, USP21, and CYLD, which have been shown to cleave M1-linked Ub chains, did not react with $Ub_{G76Dha}$-Ub (Figures 4 and 5). USP2 and USP21 are promiscuous and can cleave all types of Ub chains. The fact that they are not labeled by the OTULIN ABP may be attributed to their low activity toward M1-linkages (Bremm et al., 2010; Faesen et al., 2011; Komander et al., 2009b; Virdee et al., 2010; Ye et al., 2011). CYLD efficiently cleaves M1- or K63-linked Ub chains (Sato et al., 2015), but it still did not react with the probe. Structural comparisons of diUb binding to OTULIN or CYLD reveal that the narrow channel in CYLD, which accommodates the C-terminal tail of distal Ub and thus locates the scissile bond near the active site, may not tolerate introduction of an alanine or Dha at the position G76 (Komander et al., 2008; Renatus et al., 2006; Sato et al., 2015). The residues contacting the tail of the distal Ub are well conserved in other USPs such as USP2, USP7, USP14, and USP21 (Hu et al., 2002, 2005; Ye et al., 2011). Accordingly, USP21 and CYLD are unable to cleave the $Ub_{G76A}$-Ub substrate, indicating that Gly76 of the distal Ub is essential for recognition and cleavage of linear diUb by USPs.

Under stringent conditions, only three DUBs (OTULIN, USP5, and UCHL3) were identified by LC-MS/MS in PD experiments with bio-$Ub_{G76Dha}$-Ub (Figure 6). Remarkably, only OTULIN binding was lost after treatment with the broad-spectrum DUB inhibitor PR-619, which also inhibits USP5 and UCHL3 (Altun et al., 2011). Thus, the binding of USP5 and UCHL3 to OTULIN ABP is largely non-covalent and reversible. In contrast to the linear probe discussed here, any of the internal Lys-linked ABPs label a variety of DUBs in cell lysates (Mulder et al., 2014). This is due to the rather non-selective cleavage activity of, in particular, USP domain enzymes, of which more than 50 can be present in human cells (Komander et al., 2009a). The efficient labeling of OTULIN reflects that the OTU DUB is unique in its specific

recognition and strong catalytic activity toward M1-linked Ub chains (Mevissen et al., 2013). The non-covalent binding of OTULIN ABP to UCHL3 is most likely due to the high abundance of this enzyme. Moreover, efficient association of Ub dimers and UCHL3 without cleavage has been observed before *in vitro* and in cells (Setsuie et al., 2009). Interestingly, USP5 also largely binds reversibly to the probe, which may be due to the ability of the ZnF-ubiquitin binding domain to capture free Ub chains (Reyes-Turcu et al., 2008). However, direct DUB detection in western blots clearly shows that the OTULIN ABP can couple to USP5. It is not quite clear why the USP5-diUb product was not detected in biotin-PD assays, but structural constraints due to the large catalytic cavity of USP5 may preclude accessibility of the streptavidin beads after the attachment of biotin-diUb to USP5 (Reyes-Turcu et al., 2006). The C-terminal di-glycine motif of the most proximal Ub in a polyUb chain was shown to allosterically activate USP5 and to thereby enable cleavage of unanchored Ub chains (Reyes-Turcu et al., 2006, 2008). Indeed, by generating a probe that lacks the very C-terminal glycine residue (His-$Ub_{G76Dha}Ub_{\Delta G76}$, OTULIN ABPΔG76), we were able to eliminate cross-reactivity with USP5, yielding a DUB probe that is specific for one single enzyme of the entire DUB class of cysteine proteases.

Also, we detected proteome-wide minimal cross-reactivities of OTULIN ABP. Most striking was the energy-dependent generation of polyOTULIN ABP chains, which are detectable with an M1-specific Ub antibody. The identified ATP-dependent interaction of the E1 enzymes UBA1 or UBA6 with OTULIN ABP suggested that generation of these probe adducts is not connected to OTULIN inhibition or the enhanced conjugation of M1-linked Ub chains, as observed in OTULIN KO cells (Damgaard et al., 2016). Since the N termini of the diUbs are occupied, internal lysine residues must function as attachment sites to form unanchored branched Ub chains. Multiple lysines in the diUb probe can serve as coupling sites and accordingly USP2 and YOD1, which can cleave a number of different Ub linkages, lead to complete and partial digestion of the Ub chains, respectively. Currently, it is unclear if the finding of the *in vitro* synthesis of such artificial and branched chains is physiologically relevant, but unanchored Ub dimers and chains exist in cells and thus they may be utilized for polyUb conjugation (Chen and Pickart, 1990; Martinez-Fonts and Matouschek, 2016; Strachan et al., 2012). For diUb-based ABP, it is important to note that the auto-conjugation can be abolished by simply deleting the C-terminal Gly76, which prevents E1-catalyzed transfer.

Recently, structural studies on the K11-linkage-specific OTU DUB Cezanne demonstrated the benefits of substrate-guided DUB probes (Mevissen et al., 2016). The K11-diUb ABP reacted with the Cezanne catalytic triad enabling crystallization of an otherwise unstable and highly dynamic enzyme-substrate complex, demonstrating that the enzyme can accommodate the ABP chemistry in the active site (Mevissen et al., 2016). Here, we show how functional analyses of DUBs in cells can profit from selective probes as the OTULIN ABP (Figure 7). By nearly completely depleting substrate-bound OTULIN by biotin-PD, we show that all LUBAC subunits are associated at equivalent levels (20%–30%) with substrate-bound OTULIN. However, our data also indicate that the majority of LUBAC (~75%) is not stably binding to OTULIN in cell extracts. In line, a previous report demonstrated that a fraction of LUBAC is not associated with OTULIN (Elliott et al., 2014). It was suggested that the OTULIN-LUBAC interaction can be regulated by phosphorylation of the PIM of OTULIN (Elliott et al., 2014; Schaeffer et al., 2014), but so far we have no evidence to suggest that the association is altered upon TNF-α stimulation. Also, OTULIN activity does not change upon TNF-α stimulation. Certainly, the affinity to substrate-free OTULIN as well as $k_{on}/k_{off}$ rates may considerably influence the overall binding and regulation of LUBAC by OTULIN. However, our data strengthen the model that distinct LUBAC-DUB complexes exist and are susceptible to differential regulation, which is congruent with the observation that CYLD/SPATA2-LUBAC, but not OTULIN-LUBAC complexes, are recruited to the TNFR complex to counteract LUBAC activity post-induction (Elliott et al., 2016; Kupka et al., 2016; Schlicher et al., 2016; Wagner et al., 2016). Further, ablation of OTULIN but not CYLD was reported to promote significant accumulation of linear Ub chains in the absence of any stimulation (Damgaard et al., 2016; Draber et al., 2015).

Interestingly, effects of OTULIN deficiency are highly cell-type specific. While absence of OTULIN induces spontaneous M1-linked polyubiquitination and NF-κB activation in myeloid cells, OTULIN deficiency in T and B cells results in loss of LUBAC (Damgaard et al., 2016). The OTULIN ABP will facilitate addressing potential cell-type-specific differences in LUBAC binding and regulation by OTULIN. Moreover, our OTULIN ABP will help to characterize the physiological roles of OTULIN and in how far its functions go beyond counteracting LUBAC activity. Finally, hypomorphic mutations of OTULIN cause severe auto-inflammatory syndromes in humans (Damgaard et al., 2016; Zhou et al., 2016) and detection of active OTULIN may be used to monitor defective DUB activity *in vivo*.

## SIGNIFICANCE

**Activity-based probes (ABPs) are important tools to study enzyme activities *in vitro* and *in vivo*. Different probes for deubiquitinating enzymes (DUBs) have been introduced, but in general these probes lack selectivity, because they react with a broader panel of ubiquitin hydrolases. We used complete chemical synthesis to generate a biotinylated diUb-based probe in which the C-terminal glycine 76 of the distal ubiquitin was replaced by a Dha electrophile (bio-$Ub_{G76Dha}$-Ub). Indeed, the probe represents the first probe that recognizes and covalently labels OTULIN and thus was named OTULIN ABP. Notably, OTULIN ABP is highly selective and showed no cross-reactivity in mass spectrometry analyses. Among DUBs, the only discernible cross-reactivity was toward USP5 (Isopeptidase T), which is capable of disassembling any type of ubiquitin chain from the C terminus. Proteome-wide interaction analyses revealed ATP-dependent activation of bio-$Ub_{G76Dha}$-Ub by E1 Ub-activating enzymes UBA1 and UBA6, resulting in (auto-)polyubiquitination of the probe. Both, USP5 labeling and probe auto-conjugation are abolished by deleting glycine 76 of the proximal ubiquitin ($Ub_{G76Dha}$-$Ub_{\Delta G76}$), yielding the specific OTULIN ABPΔG76. Using our OTULIN ABP we demonstrate that OTULIN is constitutively active in cells, and that substrate-bound OTULIN associates with the linear ubiquitin chain**

**assembly complex (LUBAC). This study highlights that it is possible to generate a specific ABP for the specific linear ubiquitin hydrolase OTULIN. Overall, the here presented OTULIN ABP can be a valuable tool for assessing OTULIN activity, localization after fractionation of cellular compartments, and association with other proteins, as well as its function in physiological and pathological settings.**

## STAR★METHODS

Detailed methods are provided in the online version of this paper and include the following:

- KEY RESOURCES TABLE
- CONTACT FOR REAGENT AND RESOURCE SHARING
- EXPERIMENTAL MODEL AND SUBJECT DETAILS
  - Cell Lines
- METHOD DETAILS
  - Constructs
  - Biotin-$Ub_{G76Dha}$-Ub Synthesis
  - $His_{10}$-$Ub_{G76Dha}$-$Ub_{\Delta G76}$ Synthesis
  - Production and Purification of Recombinant Proteins
  - Analytical Size Exclusion Chromatography
  - Labeling Recombinant DUBs with OTULIN ABP or Ub-PA
  - Ubiquitin Chain Cleavage Assay
  - Crystallization
  - Structure Determination and Refinement of Crystal Structure
  - Stimulation and Transfection of Cells
  - Treatment of Cell Extracts with diUb Probes
  - Pull-down (PD) Experiments
  - Protein Digest for Mass Spectrometry Analysis
  - Liquid Chromatography- Mass Spectrometry/Mass Spectrometry (LC-MS/MS)
  - Mass Spectrometry DUB Profiling and Interactome Data Analysis
- DATA AND SOFTWARE AVAILABILITY

## SUPPLEMENTAL INFORMATION

Supplemental Information includes five figures and two tables and can be found with this article online at http://dx.doi.org/10.1016/j.chembiol.2017.08.006.

## AUTHOR CONTRIBUTIONS

A.W. conceived and performed most experiments, analyzed and interpreted the data, and helped to write the manuscript. P.E. and D.Ko. conceived and performed structural studies, contributed reagents, and provided expertise. A.P.F., S.B., and B.M.K. conceived, performed, and analyzed the MS experiments with material provided by A.W. F.E.O. designed and synthesized the bio-$Ub_{G76Dha}$-Ub probe. D.K. conceived the study/experiments, wrote the manuscript and secured funding. All authors read, acknowledged, and helped with the final version of the manuscript.

## ACKNOWLEDGMENTS

We thank Kamyar Hadian, Elisabeth Kremmer, Vishva Dixit, and Titia Sixma for providing reagents. We thank beamline staff at DLS I04 for their assistance. Access to DLS was supported in part by the EU FP7 infrastructure grant BIOSTRUCT-X (contract no. 283570). Work in the D.Ko. lab is supported by the Medical Research Council (U105192732), the European Research Council (309756), and the Lister Institute of Preventive Medicine. F.E.O. declares competing financial interests as co-founder and shareholder of UbiQ Bio BV. D.Ko. and B.M.K. are part of the DUB Alliance that includes Cancer Research Technology and FORMA Therapeutics.

Received: February 17, 2017
Revised: July 10, 2017
Accepted: August 1, 2017
Published: September 14, 2017

## REFERENCES

Altun, M., Kramer, H.B., Willems, L.I., McDermott, J.L., Leach, C.A., Goldenberg, S.J., Kumar, K.G., Konietzny, R., Fischer, R., Kogan, E., et al. (2011). Activity-based chemical proteomics accelerates inhibitor development for deubiquitylating enzymes. Chem. Biol. *18*, 1401–1412.

Bernardes, G.J., Chalker, J.M., Errey, J.C., and Davis, B.G. (2008). Facile conversion of cysteine and alkyl cysteines to dehydroalanine on protein surfaces: versatile and switchable access to functionalized proteins. J. Am. Chem. Soc. *130*, 5052–5053.

Bremm, A., Freund, S.M., and Komander, D. (2010). Lys11-linked ubiquitin chains adopt compact conformations and are preferentially hydrolyzed by the deubiquitinase Cezanne. Nat. Struct. Mol. Biol. *17*, 939–947.

Chen, Y., Wang, L., Cheng, X., Ge, X., and Wang, P. (2014). An ultrasensitive system for measuring the USPs and OTULIN activity using Nanoluc as a reporter. Biochem. Biophys. Res. Commun. *455*, 178–183.

Chen, Z., and Pickart, C.M. (1990). A 25-kilodalton ubiquitin carrier protein (E2) catalyzes multi-ubiquitin chain synthesis via lysine 48 of ubiquitin. J. Biol. Chem. *265*, 21835–21842.

Damgaard, R.B., Walker, J.A., Marco-Casanova, P., Morgan, N.V., Titheradge, H.L., Elliott, P.R., McHale, D., Maher, E.R., McKenzie, A.N., and Komander, D. (2016). The deubiquitinase OTULIN is an essential negative regulator of inflammation and autoimmunity. Cell *166*, 1215–1230.e20.

de Jong, A., Merkx, R., Berlin, I., Rodenko, B., Wijdeven, R.H., El Atmioui, D., Yalcin, Z., Robson, C.N., Neefjes, J.J., and Ovaa, H. (2012). Ubiquitin-based probes prepared by total synthesis to profile the activity of deubiquitinating enzymes. ChemBioChem *13*, 2251–2258.

Draber, P., Kupka, S., Reichert, M., Draberova, H., Lafont, E., de Miguel, D., Spilgies, L., Surinova, S., Taraborrelli, L., Hartwig, T., et al. (2015). LUBAC-recruited CYLD and A20 regulate gene activation and cell death by exerting opposing effects on linear ubiquitin in signaling complexes. Cell Rep. *13*, 2258–2272.

Duwel, M., Welteke, V., Oeckinghaus, A., Baens, M., Kloo, B., Ferch, U., Darnay, B.G., Ruland, J., Marynen, P., and Krappmann, D. (2009). A20 negatively regulates T cell receptor signaling to NF-kappaB by cleaving Malt1 ubiquitin chains. J. Immunol. *182*, 7718–7728.

Ekkebus, R., Flierman, D., Geurink, P.P., and Ovaa, H. (2014). Catching a DUB in the act: novel ubiquitin-based active site directed probes. Curr. Opin. Chem. Biol. *23*, 63–70.

Ekkebus, R., van Kasteren, S.I., Kulathu, Y., Scholten, A., Berlin, I., Geurink, P.P., de Jong, A., Goerdayal, S., Neefjes, J., Heck, A.J., et al. (2013). On terminal alkynes that can react with active-site cysteine nucleophiles in proteases. J. Am. Chem. Soc. *135*, 2867–2870.

El Oualid, F., Merkx, R., Ekkebus, R., Hameed, D.S., Smit, J.J., de Jong, A., Hilkmann, H., Sixma, T.K., and Ovaa, H. (2010). Chemical synthesis of ubiquitin, ubiquitin-based probes, and diubiquitin. Angew. Chem. Int. Ed. *49*, 10149–10153.

Elliott, P.R., Leske, D., Hrdinka, M., Bagola, K., Fiil, B.K., McLaughlin, S.H., Wagstaff, J., Volkmar, N., Christianson, J.C., Kessler, B.M., et al. (2016). SPATA2 links CYLD to LUBAC, activates CYLD, and controls LUBAC signaling. Mol. Cell *63*, 990–1005.

Elliott, P.R., Nielsen, S.V., Marco-Casanova, P., Fiil, B.K., Keusekotten, K., Mailand, N., Freund, S.M., Gyrd-Hansen, M., and Komander, D. (2014).

Molecular basis and regulation of OTULIN-LUBAC interaction. Mol. Cell *54*, 335–348.

Emsley, P., Lohkamp, B., Scott, W.G., and Cowtan, K. (2010). Features and development of Coot. Acta Crystallogr. D Biol. Crystallogr. *66*, 486–501.

Evans, P.R., and Murshudov, G.N. (2013). How good are my data and what is the resolution? Acta Crystallogr. D Biol. Crystallogr. *69*, 1204–1214.

Faesen, A.C., Luna-Vargas, M.P., Geurink, P.P., Clerici, M., Merkx, R., van Dijk, W.J., Hameed, D.S., El Oualid, F., Ovaa, H., and Sixma, T.K. (2011). The differential modulation of USP activity by internal regulatory domains, interactors and eight ubiquitin chain types. Chem. Biol. *18*, 1550–1561.

Fiil, B.K., Damgaard, R.B., Wagner, S.A., Keusekotten, K., Fritsch, M., Bekker-Jensen, S., Mailand, N., Choudhary, C., Komander, D., and Gyrd-Hansen, M. (2013). OTULIN restricts Met1-linked ubiquitination to control innate immune signaling. Mol. Cell *50*, 818–830.

Fiil, B.K., and Gyrd-Hansen, M. (2014). Met1-linked ubiquitination in immune signalling. FEBS J. *281*, 4337–4350.

Haj-Yahya, N., Hemantha, H.P., Meledin, R., Bondalapati, S., Seenaiah, M., and Brik, A. (2014). Dehydroalanine-based diubiquitin activity probes. Org. Lett. *16*, 540–543.

Hospenthal, M.K., Mevissen, T.E., and Komander, D. (2015). Deubiquitinase-based analysis of ubiquitin chain architecture using ubiquitin chain restriction (UbiCRest). Nat. Protoc. *10*, 349–361.

Hrdinka, M., Fiil, B.K., Zucca, M., Leske, D., Bagola, K., Yabal, M., Elliott, P.R., Damgaard, R.B., Komander, D., Jost, P.J., et al. (2016). CYLD limits Lys63- and Met1-linked ubiquitin at receptor complexes to regulate innate immune signaling. Cell Rep. *14*, 2846–2858.

Hu, M., Li, P., Li, M., Li, W., Yao, T., Wu, J.W., Gu, W., Cohen, R.E., and Shi, Y. (2002). Crystal structure of a UBP-family deubiquitinating enzyme in isolation and in complex with ubiquitin aldehyde. Cell *111*, 1041–1054.

Hu, M., Li, P., Song, L., Jeffrey, P.D., Chenova, T.A., Wilkinson, K.D., Cohen, R.E., and Shi, Y. (2005). Structure and mechanisms of the proteasome-associated deubiquitinating enzyme USP14. EMBO J. *24*, 3747–3756.

Iwai, K., Fujita, H., and Sasaki, Y. (2014). Linear ubiquitin chains: NF-kappaB signalling, cell death and beyond. Nat. Rev. Mol. Cell Biol. *15*, 503–508.

Keusekotten, K., Elliott, P.R., Glockner, L., Fiil, B.K., Damgaard, R.B., Kulathu, Y., Wauer, T., Hospenthal, M.K., Gyrd-Hansen, M., Krappmann, D., et al. (2013). OTULIN antagonizes LUBAC signaling by specifically hydrolyzing Met1-linked polyubiquitin. Cell *153*, 1312–1326.

Komander, D., Clague, M.J., and Urbe, S. (2009a). Breaking the chains: structure and function of the deubiquitinases. Nat. Rev. Mol. Cell Biol. *10*, 550–563.

Komander, D., Lord, C.J., Scheel, H., Swift, S., Hofmann, K., Ashworth, A., and Barford, D. (2008). The structure of the CYLD USP domain explains its specificity for Lys63-linked polyubiquitin and reveals a B box module. Mol. Cell *29*, 451–464.

Komander, D., and Rape, M. (2012). The ubiquitin code. Annu. Rev. Biochem. *81*, 203–229.

Komander, D., Reyes-Turcu, F., Licchesi, J.D., Odenwaelder, P., Wilkinson, K.D., and Barford, D. (2009b). Molecular discrimination of structurally equivalent Lys 63-linked and linear polyubiquitin chains. EMBO Rep. *10*, 466–473.

Kovalevskiy, O., Nicholls, R.A., and Murshudov, G.N. (2016). Automated refinement of macromolecular structures at low resolution using prior information. Acta Crystallogr. D Struct. Biol. *72*, 1149–1161.

Kupka, S., De Miguel, D., Draber, P., Martino, L., Surinova, S., Rittinger, K., and Walczak, H. (2016). SPATA2-mediated binding of CYLD to HOIP enables CYLD recruitment to signaling complexes. Cell Rep. *16*, 2271–2280.

Martinez-Fonts, K., and Matouschek, A. (2016). A rapid and versatile method for generating proteins with defined ubiquitin chains. Biochemistry *55*, 1898–1908.

McCoy, A.J. (2007). Solving structures of protein complexes by molecular replacement with Phaser. Acta Crystallogr. D Biol. Crystallogr. *63*, 32–41.

McGouran, J.F., Gaertner, S.R., Altun, M., Kramer, H.B., and Kessler, B.M. (2013). Deubiquitinating enzyme specificity for ubiquitin chain topology profiled by di-ubiquitin activity probes. Chem. Biol. *20*, 1447–1455.

Mevissen, T.E., Hospenthal, M.K., Geurink, P.P., Elliott, P.R., Akutsu, M., Arnaudo, N., Ekkebus, R., Kulathu, Y., Wauer, T., El Oualid, F., et al. (2013). OTU deubiquitinases reveal mechanisms of linkage specificity and enable ubiquitin chain restriction analysis. Cell *154*, 169–184.

Mevissen, T.E., Kulathu, Y., Mulder, M.P., Geurink, P.P., Maslen, S.L., Gersch, M., Elliott, P.R., Burke, J.E., van Tol, B.D., Akutsu, M., et al. (2016). Molecular basis of Lys11-polyubiquitin specificity in the deubiquitinase Cezanne. Nature *538*, 402–405.

Mulder, M.P., El Oualid, F., ter Beek, J., and Ovaa, H. (2014). A native chemical ligation handle that enables the synthesis of advanced activity-based probes: diubiquitin as a case study. ChemBioChem *15*, 946–949.

Muller-Rischart, A.K., Pilsl, A., Beaudette, P., Patra, M., Hadian, K., Funke, M., Peis, R., Deinlein, A., Schweimer, C., Kuhn, P.H., et al. (2013). The E3 ligase parkin maintains mitochondrial integrity by increasing linear ubiquitination of NEMO. Mol. Cell *49*, 908–921.

Murshudov, G.N., Skubak, P., Lebedev, A.A., Pannu, N.S., Steiner, R.A., Nicholls, R.A., Winn, M.D., Long, F., and Vagin, A.A. (2011). REFMAC5 for the refinement of macromolecular crystal structures. Acta Crystallogr. D Biol. Crystallogr. *67*, 355–367.

Renatus, M., Parrado, S.G., D'Arcy, A., Eidhoff, U., Gerhartz, B., Hassiepen, U., Pierrat, B., Riedl, R., Vinzenz, D., Worpenberg, S., et al. (2006). Structural basis of ubiquitin recognition by the deubiquitinating protease USP2. Structure *14*, 1293–1302.

Reyes-Turcu, F.E., Horton, J.R., Mullally, J.E., Heroux, A., Cheng, X., and Wilkinson, K.D. (2006). The ubiquitin binding domain ZnF UBP recognizes the C-terminal diglycine motif of unanchored ubiquitin. Cell *124*, 1197–1208.

Reyes-Turcu, F.E., Shanks, J.R., Komander, D., and Wilkinson, K.D. (2008). Recognition of polyubiquitin isoforms by the multiple ubiquitin binding modules of isopeptidase T. J. Biol. Chem. *283*, 19581–19592.

Rivkin, E., Almeida, S.M., Ceccarelli, D.F., Juang, Y.C., MacLean, T.A., Srikumar, T., Huang, H., Dunham, W.H., Fukumura, R., Xie, G., et al. (2013). The linear ubiquitin-specific deubiquitinase gumby regulates angiogenesis. Nature *498*, 318–324.

Sasaki, K., and Iwai, K. (2015). Roles of linear ubiquitinylation, a crucial regulator of NF-kappaB and cell death, in the immune system. Immunol. Rev. *266*, 175–189.

Sato, Y., Goto, E., Shibata, Y., Kubota, Y., Yamagata, A., Goto-Ito, S., Kubota, K., Inoue, J., Takekawa, M., Tokunaga, F., et al. (2015). Structures of CYLD USP with Met1- or Lys63-linked diubiquitin reveal mechanisms for dual specificity. Nat. Struct. Mol. Biol. *22*, 222–229.

Schaeffer, V., Akutsu, M., Olma, M.H., Gomes, L.C., Kawasaki, M., and Dikic, I. (2014). Binding of OTULIN to the PUB domain of HOIP controls NF-kappaB signaling. Mol. Cell *54*, 349–361.

Scharschmidt, E., Wegener, E., Heissmeyer, V., Rao, A., and Krappmann, D. (2004). Degradation of Bcl10 induced by T-cell activation negatively regulates NF-kappa B signaling. Mol. Cell. Biol. *24*, 3860–3873.

Schimmack, G., Schorpp, K., Kutzner, K., Gehring, T., Brenke, J.K., Hadian, K., and Krappmann, D. (2017). YOD1/TRAF6 association balances p62-dependent IL-1 signaling to NF-kappaB. eLife *6*, http://dx.doi.org/10.7554/eLife.22416.

Schlicher, L., Wissler, M., Preiss, F., Brauns-Schubert, P., Jakob, C., Dumit, V., Borner, C., Dengjel, J., and Maurer, U. (2016). SPATA2 promotes CYLD activity and regulates TNF-induced NF-kappaB signaling and cell death. EMBO Rep. *17*, 1485–1497.

Setsuie, R., Sakurai, M., Sakaguchi, Y., and Wada, K. (2009). Ubiquitin dimers control the hydrolase activity of UCH-L3. Neurochem. Int. *54*, 314–321.

Strachan, J., Roach, L., Sokratous, K., Tooth, D., Long, J., Garner, T.P., Searle, M.S., Oldham, N.J., and Layfield, R. (2012). Insights into the molecular composition of endogenous unanchored polyubiquitin chains. J. Proteome Res. *11*, 1969–1980.

Virdee, S., Ye, Y., Nguyen, D.P., Komander, D., and Chin, J.W. (2010). Engineered diubiquitin synthesis reveals Lys29-isopeptide specificity of an OTU deubiquitinase. Nat. Chem. Biol. *6*, 750–757.

Vizcaino, J.A., Csordas, A., del-Toro, N., Dianes, J.A., Griss, J., Lavidas, I., Mayer, G., Perez-Riverol, Y., Reisinger, F., Ternent, T., et al. (2016). 2016 update of the PRIDE database and its related tools. Nucleic Acids Res. *44*, D447–D456.

Wagner, S.A., Satpathy, S., Beli, P., and Choudhary, C. (2016). SPATA2 links CYLD to the TNF-alpha receptor signaling complex and modulates the receptor signaling outcomes. EMBO J. *35*, 1868–1884.

Wessel, D., and Flugge, U.I. (1984). A method for the quantitative recovery of protein in dilute solution in the presence of detergents and lipids. Anal. Biochem. *138*, 141–143.

Winter, G., Lobley, C.M., and Prince, S.M. (2013). Decision making in xia2. Acta Crystallogr. D Biol. Crystallogr. *69*, 1260–1273.

Ye, Y., Akutsu, M., Reyes-Turcu, F., Enchev, R.I., Wilkinson, K.D., and Komander, D. (2011). Polyubiquitin binding and cross-reactivity in the USP domain deubiquitinase USP21. EMBO Rep. *12*, 350–357.

Zhou, Q., Yu, X., Demirkaya, E., Deuitch, N., Stone, D., Tsai, W.L., Kuehn, H.S., Wang, H., Yang, D., Park, Y.H., et al. (2016). Biallelic hypomorphic mutations in a linear deubiquitinase define otulipenia, an early-onset autoinflammatory disease. Proc. Natl. Acad. Sci. USA *113*, 10127–10132.

# STAR★METHODS

## KEY RESOURCES TABLE

| REAGENT or RESOURCE | SOURCE | IDENTIFIER |
|---|---|---|
| Antibodies | | |
| Goat polyclonal anti-Actin (I-19) HRP | Santa Cruz Biotechnology | Cat#sc1616 HRP; RRID: N/A |
| Mouse monoclonal anti-CYLD (E10) | Santa Cruz Biotechnology | Cat#sc-74435; RRID: AB_1122022 |
| Mouse monoclonal anti-ubiquitin (P4D1) | Santa Cruz Biotechnology | Cat#sc-8017; RRID: AB_2315523 |
| Goat anti-biotin | Cell Signaling Technology | Cat#7075; RRID: AB_330923 |
| Rabbit polyclonal anti-OTULIN | Cell Signaling Technology | Cat#14127; RRID: AB_2576213 |
| Mouse monoclonal anti-IκBα | Cell Signaling Technology | Cat#4814; RRID: AB_390781 |
| Mouse monoclonal anti-phospho-IκBα (Ser32/36) (5A5) | Cell Signaling Technology | Cat#9246; RRID: AB_2151442 |
| Rabbit polyclonal anti-OTUB1 | Bethyl Laboratories | Cat#A302-917A; RRID: AB_10663033 |
| Rabbit polyclonal anti-USP5/IsoT | Bethyl Laboratories | Cat#A301-542A; RRID: AB_1040028 |
| Mouse monoclonal anti-FLAG M2 | Sigma-Aldrich | Cat#F3165; RRID: AB_259529 |
| Sheep polyclonal anti-HOIL-1/RBCK1 | MRC PPU Reagents and Services | Cat#S105D; RRID: N/A |
| Rabbit polyclonal anti-HOIP/RNF31 | Abcam | Cat#ab85294; RRID: AB_1925400 |
| Rabbit polyclonal anti-SHARPIN | Proteintech | Cat#14626-1-AP; RRID: AB_2187734 |
| Rabbit monoclonal anti-M1-polyUb | Millipore | Cat#MABS19; RRID:AB_2576212 |
| Human monoclonal anti-M1-polyUb | Genentech | Matsumoto et al.; RRID: N/A |
| Rat anti-HA (3F1) | Core facility monoclonal antibodies Helmholtz Zentrum München | RRID: N/A |
| Bacterial Strains | | |
| *E. coli* BL21-CodonPlus (DE3)-RIPL | Agilent Technologies | Cat#230280 |
| One Shot TOP10 Chemically Competent *E. coli* | Thermo Fisher Scientific | Cat#C404003 |
| Chemicals, Peptides, and Recombinant Proteins | | |
| Recombinant human TNF alpha | biomol | Cat#50435.50 |
| Recombinant M1-linked tetraUb chains | Enzo Life Sciences | Cat#BML-UW0785-0100 |
| Recombinant human diUb chains (K48-linked) | R&D Systems | Cat#UC-200-100 |
| Recombinant human $His_6$-CYLD Isoform 1 | R&D Systems | Cat#E-556-050 |
| Recombinant human $USP21_{cat}$ | Laboratory of Titia Sixma | (Faesen et al., 2011) |
| Recombinant human GST-YOD1 | Laboratory of Daniel Krappmann | (Schimmack et al., 2017) |
| Deconjugating Enzyme Set | R&D Systems | Cat#K-E10B |
| UbiCREST Deubiquitinase Enzyme Set | R&D Systems | Cat#K-400 |
| Biotin-Ahx-Ub-PA | UbiQ | Cat#UbiQ-076 |
| Fmoc protected amino acids | ChemImpex | |
| PyBOP | ChemImpex | Cat#02276, CAS 128625-52-5 |
| HBTU | ChemImpex | Cat#02011 CAS 94790-37-1 |
| D-Biotin | ChemImpex | Cat#00033, CAS 58-85-5 |
| 1,1,1,3,3,3-Hexafluoroisopropanol | ChemImpex | Cat#00080, CAS 920-66-1 |
| Fmoc-Gly-TentaGel Trt R resin (0.18 mmol/g) | Rapp | Cat#RA1213 |
| *N*-Methyl-2-Pyrrolidone (peptide grade) | Biosolve | Cat#13563304, CAS 872-50-4 |
| Acetonitrile (HPLC grade) | Biosolve | Cat#01201304, CAS 75-05-8 |
| Dichloromethane (S/amylene, peptide grade) | Biosolve | Cat#13793302, CAS 75-09-2 |
| *N,N*-diisopropylethylamine (peptide grade) | Biosolve | Cat#04153301, CAS 7087-68-5 |
| Water (HPLC grade) | Biosolve | Cat#23210605, CAS 7732-18-5 |
| Water (LCMS grade) | Biosolve | Cat#23217802, CAS 7732-18-5 |
| Piperidine (peptide grade) | Biosolve | Cat#16183301, CAS 110-89-4 |

(*Continued on next page*)

***Continued***

| REAGENT or RESOURCE | SOURCE | IDENTIFIER |
|---|---|---|
| Diethyl ether (AR grade) | Biosolve | Cat#05280502, CAS 60-29-7 |
| n-Pentane (AR grade) | Biosolve | Cat#16050502, CAS 109-66-0 |
| Dimethylsulfoxide (AR) | Biosolve | Cat#04470501, CAS 67-68-5 |
| Trifluoroacetic acid (peptide grade) | Biosolve | Cat#20233332, CAS 76-05-1 |
| Formic acid (99%, LCMS grade) | Biosolve | Cat#6914143, CAS 64-18-6 |
| *N,N*-Dimethylformamide (peptide grade) | Biosolce | Cat#04193301, CAS 68-12-2 |
| Triisopropylsilane (98%) | Sigma Aldrich | Cat#233781, CAS 6485-79-6 |
| Phenol (BioXtra, ≥99.5% GC) | Sigma Aldrich | Cat#P5566, CAS 108-95-2 |
| Methyl 3-mercaptopropionate (98%) | Sigma Aldrich | Cat#108987, CAS 2935-90-2 |
| 4-Mercaptophenylacetic acid (97%) | Sigma Aldrich | Cat#653152, CAS 39161-84-7 |
| HOBt | Sigma Aldrich | Cat#54802, CAS 123333-53-9 |
| *O*-mesitylenesulfonylhydroxylamine | (Bernardes et al., 2008) | CAS 36016-40-7 |
| PR-619, DUB Inhibitor V | Merck Chemicals | Cat#662141; CAS 21645-32-1 |
| cOmplete, Mini, EDTA-free Protease Inhibitor Cocktail | Roche | Cat#11836170001 |
| Pierce High Capacitiy Streptavidin Agarose | Thermo Fisher Scientific | Cat#20357 |
| Strep-Tactin Sepharose 50% suspension | IBA | Cat#2-1201-010 |
| Protino® Ni-NTA Agarose | MACHEREY-NAGEL | Cat# 745400.100 |
| Roti-Load 1, 4x conc. | Carl Roth | Cat#K929.1 |
| *E.coli* recombinant Apyrase | New England Biolabs | Cat#M0398L |
| Sequence Grade Modified Trypsin | Promega | Cat#V5111 |
| Critical Commercial Assays | | |
| Pierce Silver Stain Kit | Life Technologies | Cat#24612 |
| Deposited Data | | |
| Structure OTULIN-bio-Ub$_{G76Dha}$-Ub complex | This paper | PDB: 5OE7 |
| Structure OTULIN OTU domain (C129A) in complex with M1-linked diubiquitin | (Keusekotten et al., 2013) | PDB:3ZNZ |
| Structure zCYLD USP domain (C596S) in complex with M1-linked diubiquitin | Sato et al., 2015 | PDB: 3WXE |
| Mass spectrometry data | ProteomeXchange | PXD006868 |
| Experimental Models: Cell Lines | | |
| HEK293 cells | DSMZ | RRID: CVCL_0045 |
| Jurkat T cells | N/A | N/A |
| Recombinant DNA | | |
| pASK-IBA3(+) | IBA | Cat#2-1402-000 |
| pASK-IBA3 OTULIN$_{cat}$-Strep-tag II WT (cat: residues 80-352) | This paper | N/A |
| pASK-IBA3 OTULIN$_{cat}$-Strep-tag II C129A | This paper | N/A |
| pASK-IBA3 OTULIN$_{cat}$-Strep-tag II W96A | This paper | N/A |
| pASK-IBA3 His$_{10}$-Ub-Ub | This paper | N/A |
| pASK-IBA3 His$_{10}$-UbG76A-Ub | This paper | N/A |
| pASK-IBA3 His$_{10}$-UbG76C-UbΔG76 | This paper | N/A |
| pEF FLAG mock | Laboratory of Daniel Krappmann | (Scharschmidt et al., 2004) |
| pEF FLAG-OTULIN WT | This paper | N/A |
| pEF FLAG-OTULIN C129A | This paper | N/A |
| pcDNA3.1 (+) | Laboratory of Daniel Krappmann | (Muller-Rischart et al., 2013) |
| pcDNA3.1 HOIP-HA | Laboratory of Daniel Krappmann | (Muller-Rischart et al., 2013) |
| pcDNA3.1 HOIL-1-HA | Laboratory of Daniel Krappmann | (Muller-Rischart et al., 2013) |

(*Continued on next page*)

| *Continued* | | |
|---|---|---|
| REAGENT or RESOURCE | SOURCE | IDENTIFIER |
| pcDNA3.1 HA-SHARPIN | Laboratory of Daniel Krappmann | (Muller-Rischart et al., 2013) |
| pEF FLAG-OTUB1 | Laboratory of Kamyar Hadian | N/A |
| pEF FLAG-CYLD | Laboratory of Daniel Krappmann | N/A |
| pEF FLAG-A20 | Laboratory of Daniel Krappmann | (Duwel et al., 2009) |
| pEF FLAG-YOD1 | Laboratory of Daniel Krappmann | (Schimmack et al., 2017) |
| pEF4 FLAG-UCHL3 | Laboratory of Daniel Krappmann | N/A |
| Software and Algorithms | | |
| xia2 | (Winter et al., 2013) | http://www.ccp4.ac.uk/ |
| AIMLESS | (Evans and Murshudov, 2013) | http://www.ccp4.ac.uk/ |
| Phaser | (McCoy, 2007) | http://www.ccp4.ac.uk/ |
| COOT | (Emsley et al., 2010) | http://www.ccp4.ac.uk/ |
| Refmac5 | (Murshudov et al., 2011) | http://www.ccp4.ac.uk/ |
| PyMOL | PyMOL | http://www.pymol.org |
| MaxQuant Software (version 1.5.2) | | http://www.coxdocs.org/doku.php?id=maxquant:start |
| Perseus Software (version 1.5.5.3) | | http://www.coxdocs.org/doku.php?id=perseus:start |
| ImageJ | NIH | https://imagej.nih.gov/ij/ |
| Other | | |
| DMEM (high glucose, L-glutamine) | Life Technologies | Cat#11965092 |
| RPMI 1640 Medium (L-glutamine) | Life Technologies | Cat#21875034 |
| SOLA HRP SPE Cartridge | Thermo Scientific | Cat#60109-001 |

## CONTACT FOR REAGENT AND RESOURCE SHARING

Further information and requests for resources and reagents should be directed to and will be fulfilled by the corresponding authors, Farid El Oualid (faridelouali d@ubiqbio.com) and Daniel Krappmann (daniel.krappmann@helmholtz-muenchen.de).

## EXPERIMENTAL MODEL AND SUBJECT DETAILS

### Cell Lines

All mammalian cell lines were maintained at 37°C in a humidified atmosphere at 5% $CO_2$. Jurkat T cells were cultured in RPMI 1640 Medium, HEK293 cells in DMEM. Media were supplemented with 10% fetal calf serum, 100 U/ml penicillin and 100 μg/ml streptomycin. Jurkat T cells were authenticated by the Authentication Service of the Leibniz Institute DSMZ.

## METHOD DETAILS

### Constructs

DNA constructs for this study were generated by common molecular biological techniques using TOP10 Chemically Competent *E. Coli*. For bacterial expression of recombinant proteins, cDNAs were cloned into the pASK-IBA3(+) vector (IBA). For mammalian expression, cDNAs were cloned into a modified pEF4 (Scharschmidt et al., 2004) or the pcDNA3.1(+) backbone (Invitrogen). Mutations were introduced by site-directed mutagenesis.

### Biotin-Ub$_{G76Dha}$-Ub Synthesis

LC-MS analysis was performed on a system equipped with a Waters 2795 separation Module (Alliance HT), Waters 2996 Photodiode Array Detector (190-700 nm) and a Micromass LCT-TOF Premier mass spectrometer. Samples were run over an XBridge BEH300 C18 column (5 μm, 4.6 x 100 mm, T = 40°C) using a gradient of 30 – 60% B (over 3.5 min or 6 min). Samples were run at 0.8 mL/min using a gradient of two mobile phases: A = 1% acetonitrile and 0.1% formic acid in water; B = 1% water and 0.1% formic acid in acetonitrile. Preparative HPLC was performed on a Waters XBridge™ Prep C18 column (30 x 250 mm, 5μm OBD™). Samples were run at 25 ml/min using a gradient of two mobile phases: A = 5% acetonitrile and 0.05% trifluoroacetic acid in water; B = 5% water and 0.05% trifluoroacetic acid in acetonitrile. Gradient: 0 – 6 min: 5 – 10% B; 6 – 10 min: 10 – 30% B; 10 – 26 min: 30 – 50% B; 26 – 27 min: 50 – 95% B. Data processing was performed using Waters MassLynx 4.1 software.

### *Cys-Ub*

Full-length ubiquitin was synthesized by solid-phase peptide synthesis on Fmoc-Gly-TentaGel Trt R resin (0.18 mmol/g) as reported earlier (El Oualid et al., 2010). The resin bound Ub (25 μmol) was washed with dichloromethane (DCM) in a 20 ml syringe with a frit. Next, a solution of Fmoc-Cys(Trt)-OH (100 μmol, 58 mg) and pyBOP (125 μmol, 65 mg) in 5 ml *N*-methyl-2-pyrollidone (NMP) was added followed by *N,N*-diisopropylethylamine (DiPEA) (250 μmol, 43 μL). The mixture was mixed overnight and the resin washed with NMP and DCM. The Fmoc-Cys(Trt)-OH coupling step was repeated but this time for 1 h. After washing, the resin was treated with 10 ml of 20% piperidine in NMP for 10 min, followed by a wash with NMP and DCM. This deprotection step was repeated twice. The resin was mixed with 5 ml trifluoroacetic acid (TFA)/$H_2O$/iPr$_3$SiH/Phenol (90/5/2½/2½) for 3 h after which the TFA solution was added to 45 ml of cold diethyl ether:n-pentane 3:1 to precipitate the protein. The solution was centrifuged at 2000 rpm for 10 min (with slow brake), the diethyl ether:n-pentane decanted and 50 ml diethyl ether were added to the protein pellet. The mixture was centrifuged at 2000 rpm for 10 min (with slow brake) and after decanting the diethyl ether layer, the diethyl ether wash step was repeated. Next, the pellet was dissolved in 15 ml dimethylsulfoxide (DMSO), added to 40 ml water and purified by RP-HPLC. Lyophilization of pooled fractions afforded 46 mg of Cys-Ub as a white powder (5.3 μmol, 20%).

### *Biotin-Ahx-Ub(1-75)-SCH$_2$CH$_2$CO$_2$Me*

Ubiquitin(1-75) was synthesized by solid-phase peptide synthesis on Fmoc-Gly-TentaGel Trt R resin (0.18 mmol/g) as reported earlier (El Oualid et al., 2010). The resin bound Ub(1-75) (40 μmol) was washed with DCM in a 20 ml syringe with a frit. Next, a solution of Fmoc-aminohexanoic acid (200 μmol, 71 mg), PyBOP (200 μmol, 104 mg) and DiPEA (400 μmol, 70 μl) in 10 ml NMP was added. The syringe was sealed and the mixture mixed overnight. After washing with NMP and DCM, the Fmoc-aminohexanoic acid coupling step was repeated for 1 h. After washing with NMP and DCM, the resin was mixed with 20 ml of 20% piperidine in NMP for 10 min, followed by a wash with NMP and DCM. This deprotection step was repeated twice. After washing the resin with NMP and DCM, the resin was treated with *O*-(Benzotriazol-1-yl)-*N,N,N′,N′*-tetramethyluronium hexafluorophosphate (HBTU), D-biotin (biotin), 1-hydroxybenzotriazole (HOBt) and DiPEA. For a good dissolution of biotin, the HBTU (200 μmol, 76 mg) and HOBt (200 μmol, 31 mg) were first dissolved in 10 ml *N,N*-dimethylformamide (DMF), followed by the addition of biotin (200 μmol, 50 mg) and DiPEA (400 μmol, 70 μl). The syringe was sealed and the mixture mixed overnight. After washing with NMP and DCM, the biotin coupling step was repeated for 1 h. After washing with NMP and DCM, the biotin-Ahx-Ub(1-75) was cleaved selectively from the TentaGel Trt R resin by mixing in 30 ml of 4:1 DCM/1,1,1,3,3,3-hexafluoroisopropanol (HFIP) for 45 min. The resin was flushed 3× with 10 ml DCM and treated again with 30 ml of 4:1 DCM/HFIP for 30 min. The combined 4:1 DCM/HFIP solution was concentrated, co-evaporated 3× with DCM and dried using high vacuum. Next, the globally protected biotin-Ahx-Ub(1-75) was dissolved in 15 ml DCM and treated overnight with PyBOP (200 μmol, 76 mg), methyl 3-mercaptopropionate (200 μmol, 23 μl) and DiPEA (400 μmol, 70 μl). The DCM was evaporated and treated for 3 h with 10 ml of TFA/$H_2O$/iPr$_3$SiH/phenol (90/5/2.5/2.5 vol%). The TFA solution was added to 90 ml of cold 1:3 pentane:ether (reaction flask rinsed with 2 ml of TFA) to precipitate the protein (solution divided over two 50 ml falcon tubes). The solution was centrifuged at 2000 rpm for 10 min (with slow brake), the diethyl ether:n-pentane decanted and 100 ml diethyl ether were added to the protein pellet. The mixture was centrifuged at 2000 rpm for 10 min (with slow brake) and after decanting the ether layer, the ether wash step was repeated. The pellet was dissolved in 5 ml DMSO, added to 40 ml water and purified by RP-HPLC. Lyophilization of pooled fractions afforded 150 mg of biotin-Ahx-Ub(1-75)-SCH$_2$CH$_2$CO$_2$Me as a white powder (16.7 μmol, 42%).

### *Biotin-Ahx-Ub(1-75)-Cys-Ub*

Cys-Ub (26 mg, 3.0 μmol) and Biotin-Ahx-Ub(1-75)-S(CH$_2$)$_2$CO$_2$Me (30 mg, 3.3 μmol) were dissolved in 1 ml ligation buffer: 6M Gdn-HCl, 0.15 M NaP, 100 mM 4-mercaptophenylacetic acid (MPAA), pH 7.4 and incubated overnight at 37°C. After LC-MS analysis showing complete consumption of Cys-Ub, the reaction mixture was diluted to 20 ml with 6M Gdn-HCl, 0.15 M NaP pH 7.4 and treated with 20 mM TCEP for 30 min to reduce any disulfides. Purification by RP-HPLC and lyophilization of pooled fractions afforded 30 mg of biotin-Ahx-Ub(1-75)-Cys-Ub (1.7 μmol, 57%) as a white powder.

### *Biotin-Ahx-Ub(1-75)-Dha-Ub*

Biotin-Ahx-Ub(1-75)-Cys-Ub (30 mg, 1.7 μmol) was dissolved in DMSO (40 mg/ml, 0.75 ml) and diluted with 12 ml milliQ. Next, this was buffered with 3 ml of 500 mM sodium phosphate buffer pH 8.1 to 100 mM sodium phosphate. A solution of 6 mg *O*-mesitylenesulfonylhydroxylamine (MSH, 15 eq) (Bernardes et al., 2008; Mulder et al., 2014) in 50 μL DMF was added. After overnight incubation at 40°C, LC-MS analysis confirmed completion. Purification by RP-HPLC and lyophilization of pooled fractions afforded 21 mg biotin-Ahx-Ub(1-75)-Dha-Ub (bio-Ub$_{G76Dha}$-Ub, 1.2 μmol, 71%) (Figure S1).

For experimental applications, the probe was first dissolved to 40 mg/ml in DMSO and this was diluted to 1 mg/ml (57 μM) in 50 mM Tris (pH 7.5) and 100 mM NaCl (final DMSO concentration is 2.5 vol%).

## His$_{10}$-Ub$_{G76Dha}$-Ub$_{\Delta G76}$ Synthesis

In contrast to the OTULIN ABP synthesis, the synthesis of the His$_{10}$-Ub$_{G76Dha}$-Ub$_{\Delta G76}$ (OTULIN ABPΔG76) was not based on solid-phase peptide synthesis, but on a recombinant precursor protein. This His$_{10}$-Ub$_{G76C}$-Ub$_{\Delta G76}$, which carries a G76C substitution in the distal ubiquitin and lacks the C-terminal glycine of the proximal ubiquitin, was produced in *E. coli* BL21-CodonPlus (DE3)-RIPL cells and purified as described below. To a solution of His$_{10}$-Ub$_{G76C}$-Ub$_{\Delta G76}$ (9 ml, 0.15 mg/ml, 1.35 mg) in 100 mM sodium phosphate pH 8 was added a solution of 0.3 mg *O*-mesitylenesulfonylhydroxylamine (MSH, 15 eq) (Bernardes et al., 2008; Mulder et al., 2014) in 50 μl DMF. After overnight incubation at 40°C, LC-MS analysis showed completion (Figure S4G). For experimental applications, the probe was not further purified and stored in the present buffer (0.15 mg/ml).

## Production and Purification of Recombinant Proteins

C-terminally Strep-tag II (ST)-tagged OTULIN constructs and N-terminally $His_{10}$-tagged diUb variants were expressed in *E. coli* BL21-CodonPlus (DE3)-RIPL cells and purified via affinity chromatography using the ÄKTA Purifier system (GE Healthcare). Bacteria were grown at 37°C in LB medium containing 100 μg/ml ampicillin and 25 μg/ml chloramphenicol to an $OD_{600}$ of 0.6-0.8 before induction with 0.5 mM IPTG and 200 ng/ml anhydrotetracycline. Proteins were produced overnight at 21°C. Harvested cultures were lysed by sonification in lysis buffer (100 mM Tris pH 8.0, 150 mM NaCl, 1 mM EDTA, 0.5 mg/ml lysozyme, and protease inhibitor cocktail for ST-tagged constructs or 100 mM Tris pH 8.0, 150 mM NaCl, 30 mM imidazole, 0.5 mg/ml lysozyme, and protease inhibitor cocktail for $His_{10}$-tagged constructs). Cleared lysates containing ST-tagged proteins were applied on StrepTrap columns (GE Healthcare) and unspecifically binding proteins were washed away with washing buffer (100 mM Tris pH 8.0, 150 mM NaCl, 1 mM EDTA). Constructs were eluted using elution buffer (100 mM Tris pH 8.0, 150 mM NaCl, 1 mM EDTA, 2.5 mM D-desthiobiotin) and desalted via HiTrap Desalting columns (GE Healthcare) in 20 mM Tris pH 8, 100 mM NaCl. Cleared lysates containing $His_{10}$-tagged diUb variants were applied on HisTrap columns (GE Healthcare) and unspecific proteins were washed away with washing buffer (100 mM Tris pH 8.0, 150 mM NaCl, 30 mM imidazole). diUb constructs were eluted by a gradient of elution buffer (100 mM Tris pH 8.0, 150 mM NaCl, 300 mM imidazole) and desalted via HiTrap Desalting columns in sodium phosphate buffer pH 8.

## Analytical Size Exclusion Chromatography

Analytical size exclusion chromatography was performed on an AKTA Micro System (GE Life Sciences) using a Superdex 75 PC 3.2/30 column equilibrated in: 20 mM Tris pH 7.4, 150 mM NaCl, 2 mM DTT. In total 25 μl of 80 μM of each sample was loaded onto the column. Complexes were mixed at an equimolar ratio and incubated at room temperature for 10 min prior to loading onto the column. Fractions containing proteins were mixed with SDS sample loading buffer and subjected to SDS-PAGE analysis.

## Labeling Recombinant DUBs with OTULIN ABP or Ub-PA

Recombinant DUBs were diluted in reaction buffer (50 mM Tris, 5 mM DTT, +/- 0.03% BSA) and incubated for the indicated times at 30°C or 37°C with different amounts of OTULIN ABP or biotin-Ahx-Ub-PA (amounts specified in Figure Legends). Reactions were stopped by boiling after addition of reducing SDS sample buffer and proteins were separated by SDS-polyacrylamide gel electrophoresis (SDS-PAGE). DUB-diUb or DUB-Ub complex formation was analyzed by Coomassie staining, or Silver staining using the Pierce Silver Stain Kit according to the manufacturer's instructions.

To analyze the reactivity of OTULIN (aa 1-352) and CYLD (aa 583-956), 15 μM of each enzyme was mixed with an equimolar amount of either Ub-PA or bio-$Ub_{G76Dha}$-Ub and incubated at RT for 30 min. SDS sample buffer was used to quench the reaction and samples were resolved by SDS-PAGE analysis and stained with Coomassie.

## Ubiquitin Chain Cleavage Assay

$OTULIN_{cat}$ WT (500 pM) was diluted in reaction buffer (50 mM Tris, 5 mM DTT and 0.03% BSA) and incubated at 37°C for 25 min with 250 ng of M1-linked tetraUb chains. For inhibition, OTULIN was treated with OTULIN ABP or PR-619 at the indicated concentrations (1h, RT), prior to addition of the tetraUb chains. In diUb cleavage assays, DUBs (500 nM) were incubated in 50 mM Tris and 5 mM DTT with 500 ng of diUb chains (either K48-linked, M1-linked or $Ub_{G76A}$-Ub mutant) for 1 h at 30°C. Cleavage reactions were stopped by boiling in 1x SDS sample buffer and analyzed by Western Blot or Silver staining.

## Crystallization

The expression and purification of OTULIN (80-352) for crystallography has been described previously (Keusekotten et al., 2013). Purified OTULIN was incubated with two molar excess of bio-$Ub_{G76Dha}$-Ub (OTULIN-ABP) for 1 h at 30°C. Unreacted bio-$Ub_{G76Dha}$-Ub and OTULIN were resolved from OTULIN-diUb by anion exchange chromatography (ResourceQ; GE Healthcare) in 20 mM Tris pH 8.5, 4 mM DTT. OTULIN-diUb was further purified by size exclusion chromatography (Hiload 16/60 Superdex 75; GE Healthcare) in buffer containing 20 mM Tris pH 7.4, 150 mM NaCl, 4 mM DTT. Crystals of OTULIN-diUb were grown by sitting drop vapour diffusion around conditions that yielded the previously published OTULIN (C129A) Met1 diUb structure (1.9-2.1 M ammonium sulfate, 100 mM bis-tris pH 6.3-6.6) (Keusekotten et al., 2013). Small crystals of ~50 μm grew within 14 days and were transferred into 3.4 M sodium malonate pH 7.0 prior to cryo-cooling.

Structure determination and refinement diffraction data were collected at Diamond Light source beamline I04. Owing to the small size of the crystals, only diffraction data to 3 Å could be collected.

## Structure Determination and Refinement of Crystal Structure

Diffraction images were processed using xia2 (Winter et al., 2013) and scaled using AIMLESS (Evans and Murshudov, 2013). The cell dimensions were very similar between the OTULIN-diUb and OTULIN (C129A) Met1 diUb datasets (a=b=101.14, c= 277.92 versus a=b=100.02, c=280.26 respectively). However, the OTULIN-diUb structure converged more satisfactorily during refinement once Met1 diUb, lacking Gly75-Met1 had been placed separately to OTULIN in molecular replacement using Phaser (McCoy, 2007). Model building and refinement were performed with COOT (Emsley et al., 2010) and Refmac5 (Murshudov et al., 2011), respectively. Owing to the lower resolution of the OTULIN-diUb complex external restraints were applied from the higher resolution OTULIN (C129A) Met1 diUb structure in the early stages of refinement using PROSMART followed by separate JellyBody refinement according to

(Kovalevskiy et al., 2016) using Refmac5 (Murshudov et al., 2011). Continuous electron density could be observed for the missing dehydroalanine, which was placed with restraints generated by JLigand. Final stages of refinement included TLS parameters defined separately for OTULIN and diUb. Data collection and refinement statistics can be found in Table S1. All structure figures were generated with Pymol (www.pymol.org).

### Stimulation and Transfection of Cells

Cells were stimulated by treating them with 20 ng/ml recombinant human TNFα for 0 – 90 min. For transient overexpression of proteins, HEK293 cells were transfected using standard calcium phosphate transfection protocols.

### Treatment of Cell Extracts with diUb Probes

For the treatment of cell extracts with diUb probes, 2 – 3x$10^6$ HEK293 or Jurkat T cells were lysed in 250 μl co-IP buffer (25 mM HEPES pH 7.5, 150 mM NaCl, 0.2% NP-40, 10% glycerol, 1 mM DTT, 10 mM sodium fluoride, 8 mM β-glycerophosphate and 300 μM sodium vanadate) without protease inhibitors. Lysates were divided into aliquots (20 – 50 μl reaction volume / sample). To analyze labeling of cellular DUBs or the formation of polyUb chains, different amounts of OTULIN ABP, OTULIN ABPΔG76 or $Ub_{G76A}$-Ub were added to the samples and incubated at 30 or 37°C for 2 – 60 min (indicated in Figure Legends). Reactions were stopped by boiling in reducing sample buffer and analyzed by Western Blot. For ATP depletion, aliquots were treated with 0.5 U Apyrase for 30 min before the addition of OTULIN ABP. For linkage analyses of enriched polyUb chains, 44 μl aliquots were treated first with 1 μg OTULIN-ABP for 30 min at 37°C before adding 5 μl of different linkage-specific 10x DUBs (from the UbiCREST Deubiquitinase Enzyme Set) and incubating for further 30 min at 37°C.

### Pull-down (PD) Experiments

#### *ST-PDs*

For Strep-tag II(ST)-PDs, 20 μg of recombinant $OTULIN_{cat}$-ST (WT, C129A) were mixed with equimolar amounts of $His_{10}$-Ub-Ub WT or $His_{10}$-$Ub_{G76A}$-Ub in buffer A (PBS, 0.1% BSA, 5% glycerol, 0.1% Triton-X and protease inhibitors, total volume 500 μl) and incubated on the turning wheel for 2 h at 4°C. 20 μl were removed from each sample as Input controls (INPUT). Strep-Tactin Sepharose was pre-equilibrated in buffer A, added to the samples (60 μl of 50% slurry / sample) and incubated for 1 h with the protein mixtures to enable binding of $OTULIN_{cat}$-ST to the resin. The resin was pelleted by centrifugation (2500*g* / 4°C / 1 min) and another control sample was removed from each supernatant to check whether proteins had been depleted from the solution by the pull-down procedure. OTULIN-coupled resin was washed eight times with buffer B (PBS, 0.1% BSA, 5% glycerol, 0.5% Triton-X and protease inhibitors) to get rid of loose protein interactions and subsequently boiled in 25 μl of 2x reducing SDS sample buffer. Control samples were denatured by boiling in 1x SDS sample buffer and could be analyzed together with the pull-down eluates by Western Blot after SDS-PAGE.

#### *His-PDs / OTULIN ABPΔG76-PDs*

For His-PDs, 2x$10^7$ Jurkat T cells were lysed in lysis buffer (150 mM NaCl, 50 mM $NaH_2PO_4$, 0.2% NP-40, 10% glycerol, 10 mM imidazole, 1 mM DTT, 10 mM sodium fluoride, 8 mM β-glycerophosphate and 300 μM sodium vanadate, pH 8) without protease inhibitors for 20 min at 4°C. Cell debris was removed from the lysate by centrifugation (20,000*g*, 10 min, 4°C). To check if equal cell numbers were used, 30 μl was removed from each sample (INPUT/lysate). Cell extracts were then incubated with 4 μg $His_{10}$-$Ub_{G76Dha}$-$Ub_{\Delta G76}$ (OTULIN ABPΔG76) for 60 min at RT, enabling the coupling of active OTULIN and the probe. Ni-NTA Agarose was pre-equilibrated in lysis buffer, added to the samples (35 μl of 50% slurry per sample) and incubated on the turning wheel for 2 h at 4°C. By centrifugation (500*g* / 2 min / 4°C) beads were sedimented and subsequently washed 3 times with 1 ml of washing buffer (300 mM NaCl, 50 mM $NaH_2PO_4$, 0.2% NP-40, 10% glycerol, 20 mM imidazole, pH 8) to get rid of unspecifically binding proteins. OTULIN ABPΔG76-bound protein complexes were eluted by boiling the beads in 2x reducing SDS sample buffer (25 μl) and analyzed by Western Blot after SDS-PAGE.

#### *Biotin / OTULIN ABP-PDs*

For Biotin-PDs, 1x$10^7$ HEK293 or 2x$10^7$ Jurkat T cells were lysed in 500 μl co-IP buffer (25 mM HEPES pH 7.5, 150 mM NaCl, 0.2% NP-40, 10% glycerol, 1 mM DTT, 10 mM sodium fluoride, 8 mM β-glycerophosphate and 300 μM sodium vanadate) without protease inhibitors for 20 min at 4°C. Cell debris was removed from the lysate by centrifugation (20,000*g*, 10 min, 4°C). To check if equal cell numbers were used, 30 μl was removed from each sample (INPUT/lysate). For a pre-clearing step, cell extracts were mixed with 15 μl of High Capacitiy Streptavidin Agarose and incubated on the turning wheel for 1 h at 4°C. The beads were sedimented by centrifugation (2500*g* / 2 min / 4°C) and 450 μl of the resulting supernatant were transferred into a new reaction tube. The pre-cleared cell extracts were then incubated at with 2 - 4 μg OTULIN ABP (15 – 60 min), enabling the covalent coupling of active OTULIN and the probe. To bind and precipitate the formed OTULIN-diUb complexes, 25 – 35 μl of streptavidin agarose was added and incubated with the samples on the turning wheel for 1 - 2 h at 4°C. After a first centrifugation step (2,500*g* / 4°C / 2 min), control samples (30 μl) were removed from each supernatant to monitor depletion of proteins by the pull-down procedure. Then, beads were washed three times with 1 ml co-IP buffer to get rid of unspecific interactions. Protein complexes were eluted by boiling beads in 25 μl 2x SDS sample buffer and analyzed by Western Blot after SDS-PAGE.

To determine protein levels in input (prior to PD) and supernatant (after PD), control samples were denatured by boiling in SDS sample buffer and loaded in equal amounts on SDS gels for Western Blot Analysis. All protein band intensities were quantified using

ImageJ software and normalized to β-actin. The ratio of quantified proteins between the supernatant and input was calculated to obtain relative amounts of 'free' OTULIN-diUb unbound proteins.

To identify highly affine OTULIN ABP interactors by Mass Spectrometry Analysis, we used a larger quantity of cells ($5x10^7$ cells, lysed in 1 ml co-IP buffer) for the biotin-PDs. Pre-cleared lysates (950 μl) were incubated for 30 min at 30°C with (or without) Apyrase (4 U), to deplete cellular ATP. To inhibit DUBs, samples were incubated for further 30 min at 30°C with (or without) PR-619 (250 μM). Extracts were then treated with (or without) OTULIN ABP (5 μg) for 15 min at RT before adding 35 μl streptavidin agarose to pull-down protein-ABP complexes (2h, 4°C). In order to get rid of most non-covalent ABP interactors, beads were washed twice with co-IP buffer and subsequently twice with high-stringent 1% SDS-containing co-IP buffer. PDs were eluted by boiling the beads in 50 μl 2x SDS sample buffer. 2.5 μl (5%) was removed for Western Blot analysis, whereas the rest was analyzed by LC-MS/MS.

### Protein Digest for Mass Spectrometry Analysis

PD eluates for LC-MS/MS analysis were diluted to 175 μl with ultra-pure water and reduced with 5 μl DTT (200 mM in 0.1 M Tris, pH 7.8) for 30 min at RT. Samples were alkylated with 20 μl iodoacetamide (200 mM in 0.1 M Tris, pH 7.8) for 30 min at RT, followed by protein precipitation using a double methanol/chloroform extraction method (Wessel and Flugge, 1984). Protein samples were treated with 600 μl Methanol, 150 μl chloroform and 450 μl water, followed by vigorous vortexing. Samples were centrifuged at 17,000*g* for 3 min and the resultant upper aqueous phase was removed. Proteins were pelleted following addition of 450 μl water and centrifugation at 17,000*g* for 6 min. The supernatant was removed, and the extraction process repeated. Following the second extraction process, precipitated proteins were re-suspended in 50 μl Urea (6M) and diluted to < 1M urea with 250 μl ultra-pure water. Protein digestion was carried out with 0.6 μg trypsin (3 μl; 20 μg in 100 μl Trypsin resuspension buffer) at 37°C overnight. Following digestion, samples were acidified with 1% formic acid and desalted on C18 solid-phase extraction cartridges (SOLA HRP C18 Cartridge), dried and re-suspended in 2% acetonitrile, 0.1% formic acid for analysis by LC-MS/MS.

### Liquid Chromatography- Mass Spectrometry/Mass Spectrometry (LC-MS/MS)

LC-MS/MS analysis was performed in biological quadruplicate using a Dionex Ultimate 3000 nano-ultra high pressure reverse phase chromatography coupled on-line to a Q Exactive High Field (HF) mass spectrometer (Thermo Scientific). Samples were separated on an EASY-Spray PepMap RSLC C18 column (500 mm x 75 μm, 2 μm particle size, Thermo Scientific) over a 60 minute gradient of 2-35% acetonitrile in 5% DMSO, 0.1% formic acid at 250 nl/min. MS1 scans were acquired at a resolution of 60,000 at 200 m/z and the top 12 most abundant precursor ions were selected for HCD fragmentation.

### Mass Spectrometry DUB Profiling and Interactome Data Analysis

From raw MS files, searches against the UniProtKB human sequence data base (retrieved 15.10.2014) and label-free quantitation were performed using MaxQuant Software (v1.5.2). Search parameters include carbamidomethyl (C) as a fixed modification, oxidation (M) and deamidation (NQ as variable modifications, maximum 2 missed cleavages, matching between runs, and LFQ quantitation was performed using unique peptides. Label-free interaction data analysis was performed using Perseus (v.1.5.5.3) and volcano plots were generated using FDR=0.01 and s0=2 as cutoff parameters.

## DATA AND SOFTWARE AVAILABILITY

The structure of the OTULIN-bio-$Ub_{G76Dha}$-Ub complex has been deposited with the Protein Data Bank under the accession code PDB: 5OE7.

The mass spectrometry proteomics data have been deposited to the ProteomeXchange Consortium via the PRIDE (Vizcaino et al., 2016) partner repository with the dataset identifier PXD006868.

Cell Chemical Biology, Volume 24

## Supplemental Information

# A Linear Diubiquitin-Based Probe for Efficient and Selective Detection of the Deubiquitinating Enzyme OTULIN

**Aurelia Weber, Paul R. Elliott, Adan Pinto-Fernandez, Sarah Bonham, Benedikt M. Kessler, David Komander, Farid El Oualid, and Daniel Krappmann**

## SUPPLEMENTARY TABLES AND FIGURES

# A linear diubiquitin-based probe for efficient and selective detection of the deubiquitinating enzyme OTULIN

Aurelia Weber, Paul R. Elliott, Adan Pinto-Fernandez, Sarah Bonham, Benedikt M. Kessler, David Komander, Farid El Oualid, and Daniel Krappmann

Values in parentheses are for the highest resolution shell.

| | OTULIN 80-352-biotin-Ahx-Ub(1-75)-Dha-Ub |
|---|---|
| Data collection | |
| Beamline | Diamond I04 |
| Space group | *H* $3_2$ |
| *a*, *b*, *c* (Å) | 101.14, 101.14, 277.92 |
| $\alpha$, $\beta$, $\gamma$ (°) | 90.00, 90.00 ,120.00 |
| Wavelength | 0.9795 |
| Resolution (Å) | 54.43-2.95 (3.13-2.95) |
| $R_{merge}$ | 5.9 (70.3) |
| $< I / \sigma I >$ | 13.6 (2.0) |
| CC(1/2) | 0.99 (0.91) |
| Completeness (%) | 100 (100) |
| Redundancy | 6.4 (6.5) |
| | |
| Refinement | |
| Resolution (Å) | 92.64-2.95 |
| No. reflections | 11911 |
| $R_{work}$ / $R_{free}$ | 0.22/0.32 |
| No. atoms | 3104 |
| Protein | 3104 |
| Ligand/ion | - |
| Water | - |
| *B*-factors | |
| Wilson B | 93.98 |
| Protein | 77.08 |
| Ligand/ion | - |
| Water | - |
| R.m.s deviations | |
| Bond lengths (Å) | 0.018 |
| Bond angles (°) | 1.955 |
| Ramachandran statistics (outliers, allowed, favoured) | |

Dataset was collected from a single crystal.

**Table S1 (related to Fig. 3):** Data collection and refinement statistics for crystallography.

| | | LOG2 LFQ intensities | | | | | | | | | | | | | | | | | | | | | |
|---|---|---|---|---|---|---|---|---|---|---|---|---|---|---|---|---|---|---|---|---|---|---|---|
| | | No Probe | | | | OTULIN-APB | | | | OTULIN-ABP + Apyrase | | | | OTULIN-ABP + Apyrase + PR619 | | | | Significance difference No Probe_OTULIN-ABP (Student's T-test) | | | | | |
| Protein IDs | Protein names | LFQ I 1_1 | LFQ I 1_2 | LFQ 1_3 | LFQ I 1_4 | LFQ I 2_1 | LFQ I 2_2 | LFQ I 2_3 | LFQ I 2_4 | LFQ 3_1 | LFQ I 3_2 | LFQ I 3_3 | LFQ I 3_4 | LFQ I 4_1 | LFQ I 4_2 | LFQ I 4_3 | LFQ I 4_4 | | N: -Log STT | N: STT | N: STT statistic | MW [kDa] | Score |
| P22314-2;P22314 | UBA1 | 17,7766 | 17,5063 | 17,5048 | 17,707 | 29,2205 | 29,5495 | 29,7971 | 30,1985 | 23,5485 | 21,5931 | 19,6165 | 20,4098 | 21,8335 | 20,1046 | 20,6147 | 19,3192 | + | 8,63627 | -12,0677 | -5,4417 | 113,8 | 323,31 |
| Q96BN8;H0Y9T0 | OTULIN | 17,1191 | 16,7217 | 15,4617 | 16,6438 | 29,0197 | 29,4706 | 29,7409 | 29,2958 | 29,8383 | 29,3178 | 28,8216 | 28,1979 | 23,6984 | 23,434 | 22,1332 | 22,3256 | + | 7,30689 | -12,8952 | -5,40014 | 40,262 | 308,75 |
| P15374;Q5TBK7 | UCHL3 | 17,7447 | 17,6595 | 15,7178 | 15,9059 | 24,2089 | 25,0379 | 24,8467 | 24,4435 | 25,0074 | 25,0494 | 24,3842 | 24,4533 | 25,1512 | 25,7674 | 24,9247 | 24,739 | + | 5,01022 | -7,87727 | -3,05465 | 26,182 | 62,597 |
| A0AVT1;A0AVT1-2 | UBA6 | 18,5863 | 16,5304 | 17,4451 | 17,8637 | 22,7336 | 23,4294 | 24,0412 | 24,2625 | 17,2079 | 18,4227 | 17,7002 | 16,9856 | 18,042 | 17,8221 | 17,6305 | 17,2594 | + | 4,4604 | -6,0103 | -2,35751 | 117,97 | 75,662 |
| F8VSA6;E9PS38 | NEDD8 | 17,8619 | 17,5603 | 15,351 | 15,947 | 22,6509 | 23,4459 | 24,6994 | 23,244 | 23,8412 | 23,2567 | 23,1742 | 23,0965 | 24,4478 | 24,3355 | 24,1069 | 24,2666 | + | 4,0151 | -6,82999 | -2,48599 | 5,8668 | 21,287 |
| P45974-2;P45974 | USP5 | 17,798 | 20,1282 | 15,5395 | 20,1224 | 26,2123 | 27,7202 | 26,3578 | 26,3365 | 27,9555 | 26,4718 | 26,66 | 26,1792 | 26,3028 | 26,0168 | 25,6588 | 25,687 | + | 3,42288 | -8,25972 | -2,61767 | 93,307 | 154,3 |
| F5H265;J3QS39 | UBC;UBB | 23,6918 | 24,1324 | 16,9545 | 16,6629 | 29,8302 | 30,9425 | 31,9719 | 31,6184 | 30,5698 | 31,2093 | 30,1123 | 30,8753 | 30,7342 | 30,8444 | 29,8138 | 31,1985 | + | 2,65046 | -10,7303 | -2,61282 | 16,841 | 323,31 |

**Table S2 (related to Fig. 6):** DUB enzymes identified as OTULIN ABP interactors by LC-MS/MS after biotin-PD. Perseus Label-free interaction data analysis workflow was performed using the following cutoff parameters: FDR = 0.01 and s0 = 2. LFQ intensities are given for each sample. Significance was calculated by Student's T test (STT).

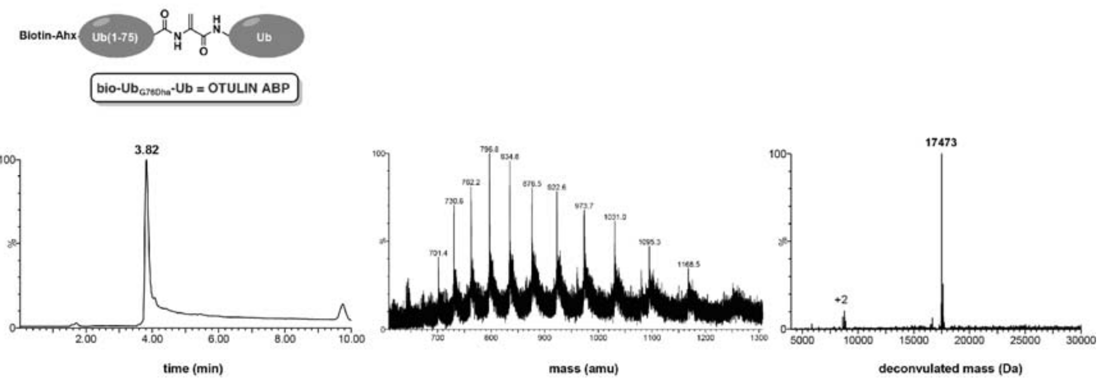


**Figure S1 (related to Fig. 1):** LC-MS analysis Biotin-Ahx-Ub(1-75)-Dha-Ub (bio-Ub$_{G76Dha}$-Ub).

Mobile phase A = 1% $CH_3CN$, 0.1% formic acid in water (milliQ) and B = 1% water (milliQ) and 0.1% formic acid in $CH_3CN$. XBridge BEH300 C18 5µm 4.6x100mm; column T = 40°C, flow= 0.8 mL/min. Gradient: 0 - 1.5 min: 5-30% B; 1.5 - 8 min: 30-50% B; 8 – 9.5 min: 50-95% B.

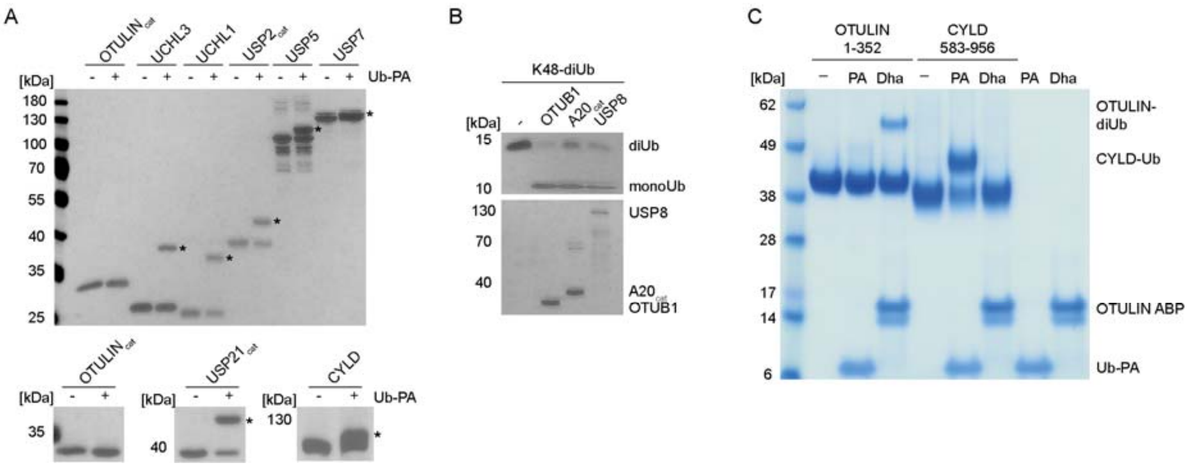


**Figure S2 (related to Figure 4)**: Validation of DUB activities

A) Validation of DUB activities with Ub-PA probe. Recombinant DUBs (0.5 µM) were incubated at 30°C with biotin-Ahx-Ub-PA probe (5 µM) for 1 h. Labeling was monitored by Silver staining. Asterisks denote the DUB-Ub adducts.

B) Validation of DUB activities by K48-diUb cleavage assay. OTUB1, $A20_{cat}$ and USP8 (0.5 µM) were incubated with 500 ng of K48-linked diUb at 30°C for 1 h before analyzing the diUb cleavage by Silver staining.

C) Direct comparison of OTULIN and CYLD labeling by OTULIN ABP and Ub-PA. Recombinant OTULIN (aa 1-352) or CYLD (aa 583-956), both at 30 µM were mixed with equimolar Ub-PA (PA) or bio-$Ub_{G76Dha}$-Ub (Dha) and incubated at RT for 1 h. Samples were resolved by SDS-PAGE and stained with Coomassie.

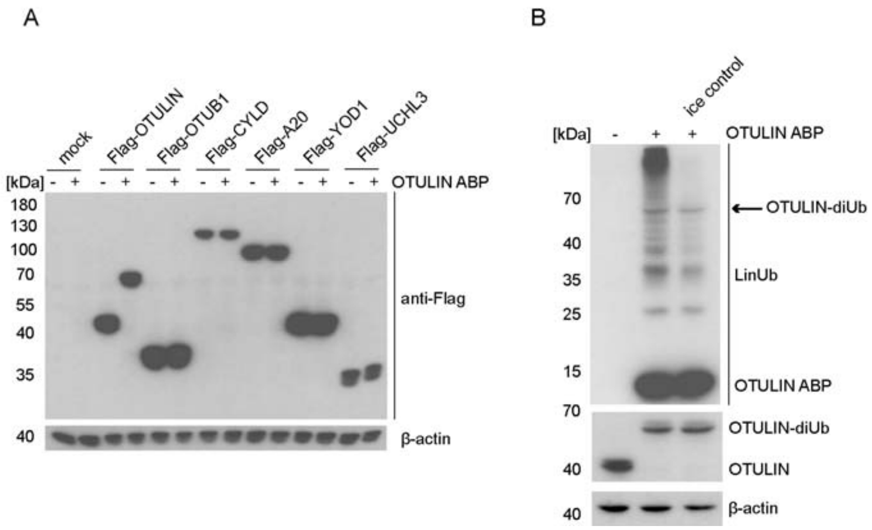


**Figure S3 (related to Fig. 5):** Labeling of overexpressed DUBs and induced Ub chain synthesis by OTULIN ABP.

A) Cross-reactivity of OTULIN ABP with transfected DUBs in HEK293 cells. Cell extracts from HEK293 cells overexpressing Flag-tagged DUBs (~$2.5*10^5$ cells / reaction) were incubated with OTULIN ABP (30°C, 1 h). Labeling of DUBs was analyzed by Western Blot using anti-Flag antibody.

B) Temperature-dependent polyUb chain formation induced by OTULIN ABP. HEK293 cell extracts (~$2.5*10^5$ cells / reaction) were mixed with 1 µg OTULIN ABP and incubated for 30 min at 30°C. In the control lane, the enzymatic reaction was put on ice after an initial 2 min incubation at 30°C. Formation of the OTULIN-diUb adducts and linear ubiquitin chains were monitored by Western Blot.

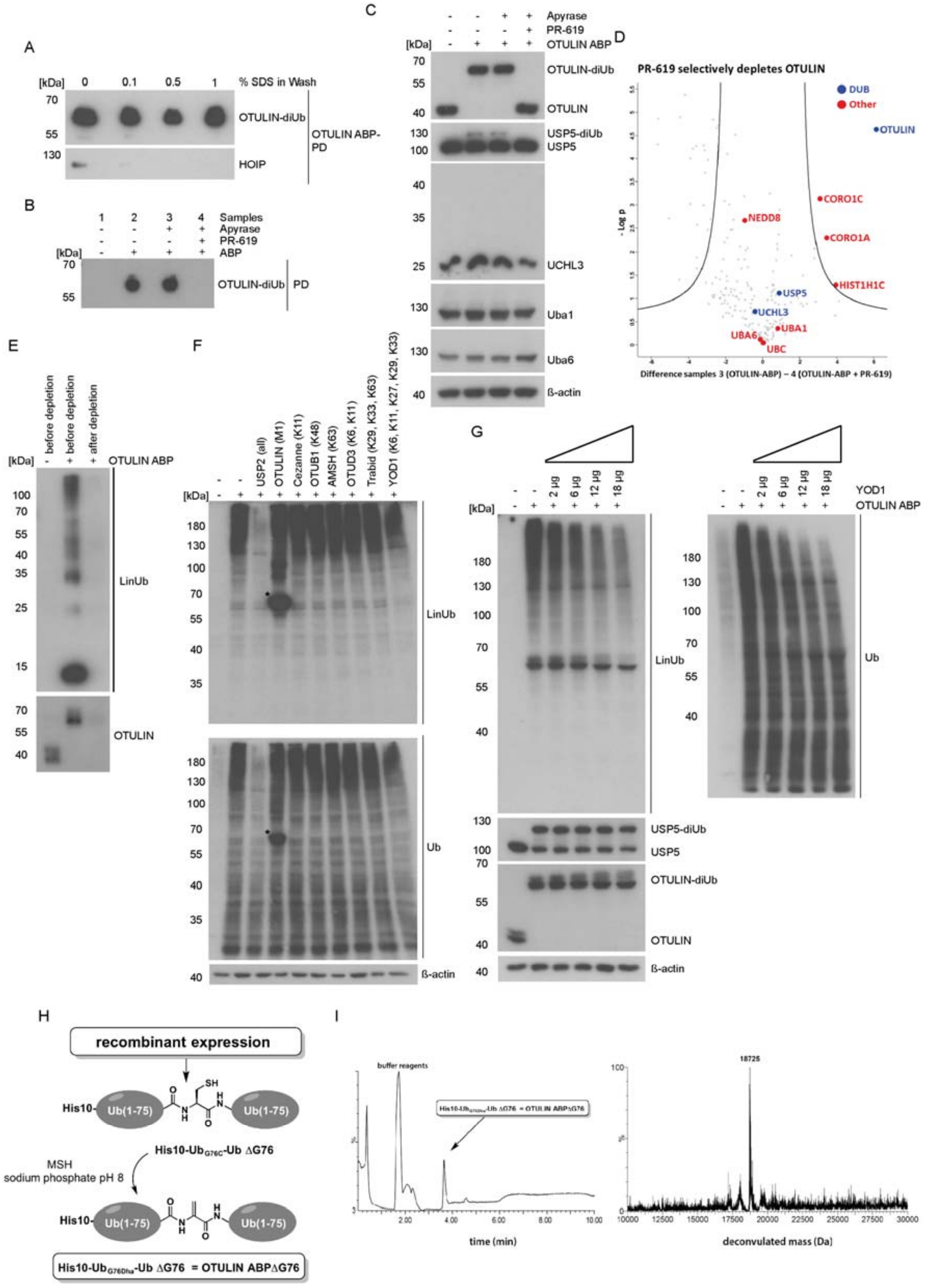


**Figure S4 (related to Fig. 6):** Specificity of probe labeling and auto-conjugation; synthesis of OTULIN ABPΔG76

A) Biotin-PD of OTULIN-diUb complex under high stringent washing conditions. 4 µg of OTULIN ABP were incubated in extracts of Jurkat T cells ($18*10^6$ cells per reaction) for 15 min at RT. After PD, beads were washed twice with increasing SDS concentrations to enrich for covalent ABP interactors. Pull-down eluates were analyzed for OTULIN-diUb complex and HOIP by Western Blot.

B) Control of OTULIN-diUb pull-down in samples before LC-MS/MS analyses. Cell extracts of $5x10^7$ Jurkat T cells were subjected to treatments (as indicated; see Fig. 6A) and biotin-PD including 1% SDS washes was performed. PD of OTULIN-diUb complex was verified by analyzing 5% of the eluted material that was subsequently used for LC-MS/MS analyses.

C) OTULIN ABP labeling in extracts of Jurkat T cells. Extracts of Jurkat T cells were treated equivalent to LC-MS/MS (see Fig. 1A) and coupling of OTULIN ABP to DUBs and E1 enzymes in the extracts was determined by Western Blot.

D) Interactome analyses reveal selective depletion of OTULIN and HOIP from the OTULIN ABP by PR-619 treatment. Volcano plot demonstrates loss of binding of DUBs (blue) and other proteins (red) between control (sample 3) and PR-619 (sample 4) treatments before OTULIN ABP incubation and biotin-PD. Curves depict significant enrichment or depletion, respectively. Loss of COR1C, FHL2 and TPM3 is only associated with PR-619 treatment and not with specific OTULIN ABP interaction.

E) OTULIN ABP is auto-conjugated in cell extracts. Extracts of Jurkat T cells ($6*10^5$ cells / reaction) treated with 1 µg OTULIN ABP (45 min, 30°C) were subjected to biotin-PD. Depletion of Ub chains was demonstrated by comparing presence of Ub chains in the extracts before and after biotin-PD. Depletion of OTULIN-diUb adduct is shown as control.

F) OTULIN ABP induces generation of Ub chains that are prone to USP2 digestion. Linkage analyses of Ub chains formed in extracts of HEK293 cells after OTULIN ABP incubation (30 min at 37°C) were performed by UbiCrest digestion. Treatment with linkage-specific DUBs was performed for another 30 min. Ubiquitin chains were detected by Western Blotting with anti-M1-polyUb or anti-Ub antibodies. Asterisk indicates formation OTULIN-diUb adduct by the OTULIN ABP.

G) High concentrations of YOD1 lead to partial digestion of OTULIN ABP induced Ub chains. Increasing YOD1 amounts were added (30 min) to extracts of HEK293 cells Ub chains were generate by OTULIN ABP incubation as in D. Formation of polyUb chains was detected by Western Blotting with anti-M1-polyUb or anti-Ub antibodies.

H) Chemical synthesis of His-$Ub_{G76Dha}$-$Ub_{\Delta G76}$ (OTULIN ABP$\Delta$G76)

I) LC-MS analysis His-Ub(1-75)-Dha-Ub(1-75) (His-$Ub_{G76Dha}$-$Ub_{\Delta G76}$)

Figure S5 (related to Fig. 7)

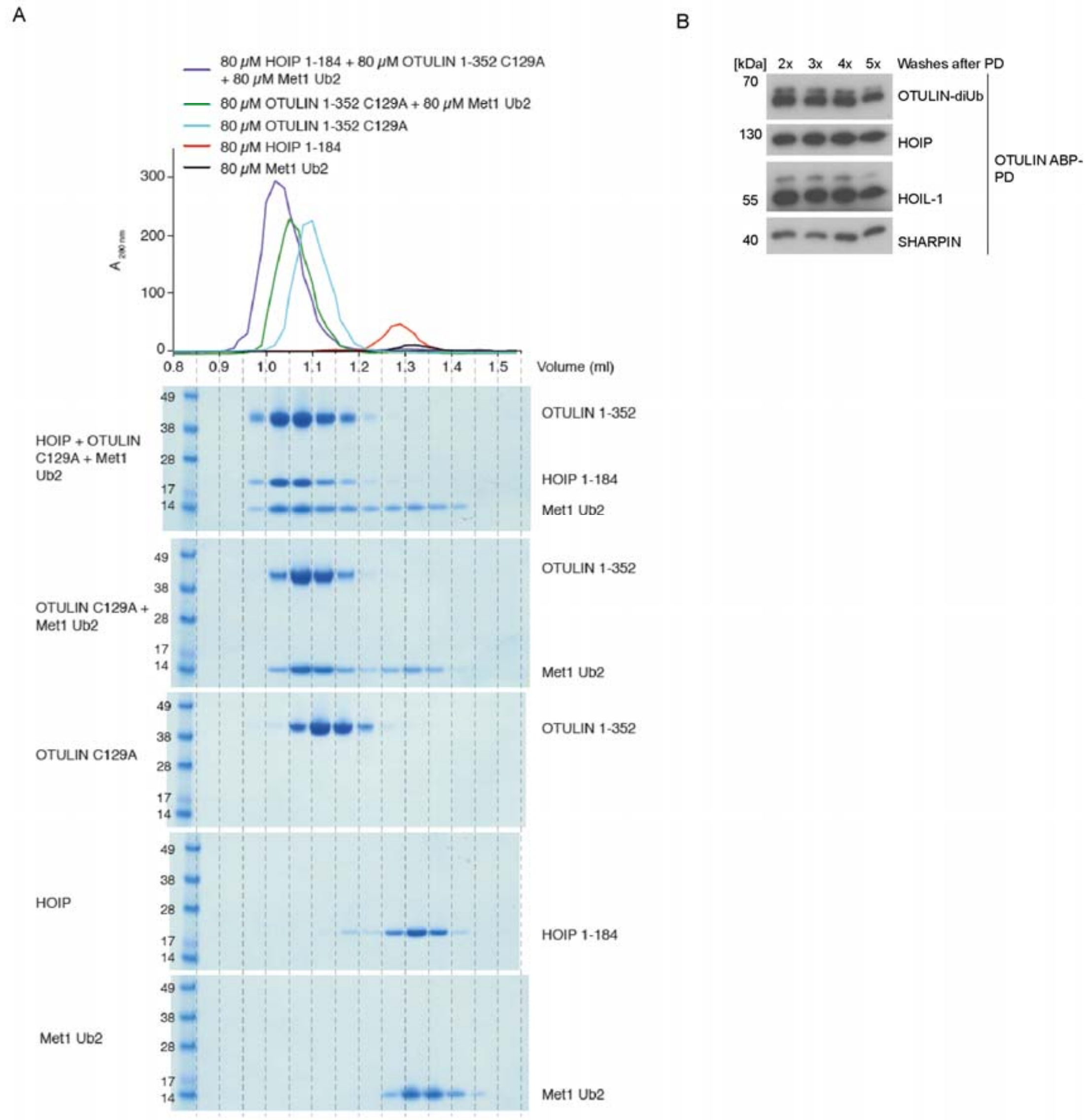


**Figure S5 (related to Fig. 7):** Data on stability of ternary OTULIN-diUb-LUBAC interaction *in vitro* and from cells

A) Size exclusion chromatography demonstrates ternary complex of HOIP, OTULIN and M1-linked diUb in vitro. Analytical size exclusion chromatography profiles for OTULIN (aa 1-352; C129A; cyan), HOIP PUB domain (aa 1-1184; red), M1-diUb (black curve), and the dimeric complex between OTULIN and M1-diUb (green curve) and trimeric complex between HOIP, OTULIN and M1-diUb (blue curve) at equimolar ratios. Coomassie-stained SDS-PAGE gels below show protein-containing fractions.

B) Stability of the OTULIN-diUb product bound to the cellular LUBAC in extracts. Extracts of Jurkat T cells were incubated with OTULIN ABP and biotin-PDs were performed as in Fig. 7C. Stability of the LUBAC association to the OTULIN-diUb product was monitored by analyzing successive (2x – 5x) washing steps of the pull-downs.
